# Assessment of the in vitro anti-diabetic activity with molecular dynamic simulations of limonoids isolated from Adalia lemon peels

**DOI:** 10.1038/s41598-024-71198-5

**Published:** 2024-09-14

**Authors:** Amal M. El-Feky, Wael Mahmoud Aboulthana, Ahmed A. El-Rashedy

**Affiliations:** 1 Pharmacognosy Department, Pharmaceutical and Drug Industries Research Institute, National Research Centre, 33 El Bohouth St. (Former El Tahrir St.), P.O. 12622, Dokki, Giza, Egypt; 2 Biochemistry Department, Biotechnology Research Institute, National Research Centre, 33 El Bohouth St. (Former El Tahrir St.), P.O. 12622, Dokki, Giza, Egypt; 3 Natural and Microbial Products Department, Pharmaceutical and Drug Industries Research Institute, National Research Centre, 33 El Bohouth St. (Former El Tahrir St.), P.O. 12622, Dokki, Giza, Egypt

**Keywords:** Adalia lemon, Limonoids, Scavenging activity, Anti-diabetic activity, Molecular dynamic simulation, Biochemistry, Chemical biology

## Abstract

Limonoids are important constituents of citrus that have a significant impact on promoting human health. Therefore, the primary focus of this research was to assess the overall limonoid content and isolate limonoids from Adalia lemon (*Citrus limon* L.) peels for their potential use as antioxidants and anti-diabetic agents. The levels of limonoid aglycones in the *C. limon* peel extract were quantified through a colorimetric assay, revealing a concentration of 16.53 ± 0.93 mg/L limonin equivalent. Furthermore, the total concentration of limonoid glucosides was determined to be 54.38 ± 1.02 mg/L. The study successfully identified five isolated limonoids, namely limonin, deacetylnomilin, nomilin, obacunone 17-O-β-D-glucopyranoside, and limonin 17-O-β-D-glucopyranoside, along with their respective yields. The efficacy of the limonoids-rich extract and the five isolated compounds was evaluated at three different concentrations (50, 100, and 200 µg/mL). It was found that both obacunone 17-O-β-D-glucopyranoside and limonin 17-O-β-D-glucopyranoside possessed the highest antioxidant, free radical scavenging, and anti-diabetic activities, followed by deacetylnomilin, and then the limonoids-rich extract. The molecular dynamic simulations were conducted to predict the behavior of the isolated compounds upon binding to the protein's active site, as well as their interaction and stability. The results revealed that limonin 17-O-β-D-glucopyranoside bound to the protein complex system exhibited a relatively more stable conformation than the Apo system. The analysis of Solvent Accessible Surface Area (SASA), in conjunction with the data obtained from Root-Mean-Square Deviation (RMSD), Root-Mean-Square Fluctuation (RMSF), and Radius of Gyration (ROG) computations, provided further evidence that the limonin 17-O-β-D-glucopyranoside complex system remained stable within the catalytic domain binding site of the human pancreatic alpha-amylase (HPA)-receptor. The research findings suggest that the limonoids found in Adalia lemon peels have the potential to be used as effective natural substances in creating innovative therapeutic treatments for conditions related to oxidative stress and disorders in carbohydrate metabolism.

## Introduction

Agronomic fruits and vegetables produce a significant amount of agricultural by-products. If properly utilized, these abundant remnants can have considerable importance^1^. In Egypt, *citrus* fruits have been cultivated since ancient times and continue to be one of the primary crops^2^. The *citrus* handling manufactures produce a staggering 15 million tons of waste each year, which mainly consists of peels and seeds^3^. These leftover *citrus* materials are rich in bioactive compounds and have immense potential for the creation of cutting-edge natural products, offering an effective, cost-efficient, and environmentally friendly avenue^4^.

In recent times, there has been a noticeable surge in the utilization of fruit peels in various ways, which not only aids in the reduction of solid waste management but also enhances the value of these discarded peels^5,6^. This is achieved by transforming the materials derived from fruit wastes into valuable natural products that can serve as viable alternative medicines^7,8^. The attractiveness of these byproducts lies in their accessibility, and cost-effectiveness when compared to modern therapeutic drugs^9^.

Adalia lemons, *Citrus limon* L., are highly cultivated fruits globally. As a member of the Rutaceae family, Adalia lemons are of great significance in the medicinal field as they play a vital role in enhancing human well-being and nourishment^10^. Numerous studies have focused on exploring the extensive pharmacological actions of the peels removed from *C. limon* fruits^11^. The utilization of lemon peels has been extensively studied by scholars. These investigations have revealed that *C. limon* peels possess notable antimicrobial effects and exhibit astringent properties^12,13^. Furthermore, they have been found to be effective in managing urolithiasis and reducing liver and plasma cholesterol levels. Additionally, the utilization of the peel extract has demonstrated favorable outcomes in addressing the issue of childhood obesity^14,15^.

Limonoids, coumarins, and flavonoids represent the primary phytoconstituents found in *citrus* peels^16^. These compounds play a crucial role in promoting human health by exhibiting various biological activities, including antioxidative, cytotoxic, antimutagenic, anti-inflammatory, antiulcer, and antiviral properties^10,17^. Limonoids, a distinctive group of highly oxygenated tetranortriterpenoids, are responsible for the characteristic bitter taste found in *citrus* fruits^18^. Over the past few decades, there has been a growing interest in studying *Citrus* limonoids due to their diverse range of bioactivities^19,20^, such as antitumor, antimicrobial, antioxidant and antiviral properties^21,22^. According to previous studies, *Citrus* limonoids have demonstrated promising bioactivities, making them potentially valuable in the food and pharmaceutical sectors^23,24^. Consequently, there is a growing need for an efficient and cost-effective method to extract and purify these bioactive compounds.

A molecular dynamics simulation was carried out to predict the performance of the isolated compounds upon binding to the active site of the protein, as well as their interaction and stability through simulation^25^. Assessing protein structural flexibility upon ligand binding is critical for examining residue behavior and its connection with the ligand^26^. One of the purposes of drug design is to make structural changes to therapeutic molecules to increase bioavailability, reduce toxicity, and improve pharmacokinetics^27^. This research was designed to isolate and explore the potential applications of limonoids from low-cost agricultural waste material, especially Adalia lemon peels. Specifically, the focus was on investigating the antioxidant and anti-diabetic properties of these limonoids, highlighting their untapped potential as valuable resources. Additionally, the inhibitory impacts of these limonoids on both free radicals and enzymes involved in carbohydrate metabolism were predicted by molecular dynamic simulations.

## Materials and methods

### Preparation of plant extract

Fresh Adalia lemons were harvested in January 2024 from a privately owned farm in El-Slaheya Elgdeda, Sharkia Governorate, Egypt. The taxonomical identification was conducted by Eng. Therese Labib, a Consultant of plant taxonomy at the Ministry of Agriculture in Giza, Egypt. A specimen was deposited in the herbarium of the National Research Centre (NRC) in Cairo, Egypt (Voucher No. M221).

The lemon peels underwent a process of air drying in a shaded area. Subsequently, the resulting dried lemon peel powder weighing 300 g was extracted consecutively using hexane (4 × 750 mL) to remove the fatty matter from the lemon peels. After filtering the collected extract, it was evaporated under reduced pressure, resulting in a fatty residue weighing 5 g. Subsequently, the extraction process continued using a solvent system consisting of acetone and ethyl acetate in a 1:1 ratio. The selection of this specific solvent combination was determined by the findings presented in the research paper authored by Magurano et al.^28^. Using a rotary evaporator, the obtained extract was concentrated at a temperature of 40 °C, resulting in a yield of 9 g.

### Determination of total limonoids content

The assessment of the total limonoids content involved a colorimetric assay for both limonoidaglycones and glucosides, following the established protocol outlined in the study conducted by Breksa and Ibarra^29^. The results were expressed as limonin equivalents, with the mean value presented alongside the standard error (SE) calculated from triplicates of experimental measurements.

### Isolation of limonoids

For column chromatography, a limonoid-rich extract of *C. limon* peels (5 g) was subjected to 60 g of silica gel in a column measuring 1.5 × 30 cm. The elution process involved a gradient of ethyl acetate, with the polarity progressively increased through the addition of methanol. Thin-layer chromatography (TLC) investigation was performed on all collected fractions using a silica gel GF254 plate Aluminum sheets 20X20, layer thickness 0.2 mm (Merck) and a solvent mixture of chloroform and methanol (9.5:0.5) for development. The resulting spots were visualized by spraying with a mixture of vanillin and H_2_SO_4_, followed by heating at 120 oC for 3 min. The fractions that produced similar characteristics were merged together and subsequently purified on TLC using a mixture of ethyl acetate, methanol, and water in a 6:1:0.6 ratio. This process led to the isolation of compound 1 (32 mg, R_*f*_ 0.86) from the fraction composed of ethyl acetate and methanol (in a ratio of 95:5). From the fraction containing ethyl acetate and methanol in a ratio of 90:10, compounds 2 (28 mg, R_*f*_ 0.75) and 3 (19 mg, R_*f*_ 0.69) were obtained. Compounds 4 (21 mg, Rf 0.53) and 5 (16 mg, Rf 0.47) were isolated from fractions with ratios of 80:20 and 75:25, respectively. To determine the structures of these compounds, various techniques including EI-MS (Finnigan Model 3200 Mass spectrometer), ^1^H-NMR, and ^13^C-NMR (JEOL EX- 500 MHz, Japan) were employed.

### In vitro biological activities

All in vitro biological activities of the limonoids-rich extract and the isolated compounds were assayed in triplicate at three different concentrations (50, 100, and 200 µg/mL).

#### Antioxidant activity

The method proposed by Prieto et al.^30^ was used to determine the total antioxidant capacity (TAC) expressed as mg gallic acid equivalent per gram weight, using ascorbic acid at the same concentrations as a standard. The method demonstrated by Oyaizu^31^ was used to assess the iron reducing power (IRP) expressed as µg/mL, using ascorbic acid as a standard.

#### Scavenging activity

The 1,1-Diphenyl-2-picryl-hydrazyl (DPPH) radical scavenging activity was evaluated using the method described by Rahman et al.^32^. Ascorbic acid was used as a positive control at the same concentrations. The inhibition percentage of the DPPH free radical was calculated. The median inhibitory concentration (IC_50_) of each tested sample was determined by plotting a curve using a series of sample concentrations against the percentage of DPPH inhibition.

The procedure for the 2,2'-azinobis-(3-ethylbenzothiazoline-6-sulfonic acid) (ABTS) assay followed the method suggested by Arnao et al.^33^. The ABTS scavenging capacities of the samples were compared with that of ascorbic acid. The percent inhibition of the ABTS radical was calculated. The IC_50_ of each tested sample was determined by plotting a curve using a series of sample concentrations against the percent of ABTS inhibition.

The nitric oxide (NO) radical scavenging activity was estimated by the reaction with Griess Illosvory reagent based on the method proposed by Chakraborthy^34^. The NO scavenging capacities of the samples were compared with that of ascorbic acid. The percent inhibition of the NO radical was calculated. The IC_50_ of each tested sample was determined by plotting a curve using a series of sample concentrations against the percent of NO inhibition.

#### Anti-diabetic activity

The enzyme assay involved calculating the inhibition percentage (%) of α-amylase^35^ and α-glucosidase enzymes^36^ with acarbose as the standard drug. The IC_50_ of each tested sample was determined by plotting a curve using a series of sample concentrations against the percent of enzyme inhibition.

The native electrophoretic α-amylase isoenzyme pattern was assayed using vertical slab polyacrylamide gel electrophoresis (PAGE) following the method suggested by Rammesmayer and Praznik^37^. The sodium dodecyl sulfate (SDS) electrophoretic α-glucosidase pattern was conducted following the method suggested by Laemmli^38^ using Mini-gel electrophoresis (BioRad, USA) to determine the activity of the α-glucosidase enzyme which was checked and visualized by Coomassie Brilliant Blue (CBB), and compared to acarbose (standard).

The relative mobility (Rf), band quantity (Qty) and band percent (B%) of the electrophoretically detected isoenzyme types on the PAGE plate were determined using the Quantity One software program (version 4.6.2). The equation proposed by Nei and Li^39^ was used to calculate the percentages of similarity index (SI%) and physiological difference (Diff%).

#### Statistical analysis

A one-way analysis of variance (one-way ANOVA) was performed using the Statistical Package for the Social Sciences (SPSS for Windows, version 11.0) to evaluate the statistically significant correlations among the different in vitro biological measurements. The correlation was considered significant at *P* < 0.05 and highly significant at *P* < 0.01.

### Molecular docking and molecular dynamic (MD) simulations

#### System preparation and molecular docking

Using codes 1B2Y^40^, the 3D structures of human pancreatic alpha-amylase (HPA) in complex with their competitive inhibitor acarbose were obtained from the Protein Data Bank and constructed using UCSF Chimera^41^. pH was adjusted and tuned to 7.5 using PROPKA^42^. Using ChemBioDraw Ultra 12.1, the derived 2D structure was shown^43^. The Avogadro program^44^ was utilized to optimize 2D structure for energy minimization, employing the steepest descent approach and MMFF94 force field. UCSF chimaera was used to remove hydrogen atoms prior to docking^41^.

The docking computations were performed using AutoDockVina^45^, and the docking process included the allocation of Gasteiger partial charges^46^. To outline the AutoDock atom types, the MGL tools AutoDock graphical user interface was utilized. The size of the search space was set to 20 Å × 20 Å × 20 Å, with exhaustiveness set to 8. The AutoDockVina grid center coordinates used are 16.86 Å, 0.99 Å, and 45.73 Å for x, y, and z. Docked conformations were generated in descending order according to their docking energy using the Lamarckian genetic method^47^.

#### Molecular dynamic (MD) simulations

ANTECHAMBER's General Amber Force Field (GAFF) method was used to determine each compound's partial atomic charge^48^. Within an orthorhombic box of TIP3P water molecules, each system was implicitly solvated within 10 Å of any box edge by the Leap module of the AMBER 18 package. Na + and Cl-counter ions were added to each system using the Leap module to neutralize it. Each system underwent a 2000-step initial minimization with an imposed restraint potential of 500 kcal/mol, and a 1000-step full minimization with the conjugate gradient algorithm in the absence of constraints.

To guarantee that every system had the same number of atoms and volume throughout the MD simulation, each system was heated incrementally over 500 ps, from 0 to 300 K. The solutes in the system were subjected to a collision frequency of 1 ps and a potential harmonic constraint of 10 kcal/mol. Every system was then heated to a constant temperature of 300 K and allowed to equilibrate for 500 ps.The number of atoms and pressure within each system were kept constant for each production simulation in order to model an isobaric-isothermal (NPT) ensemble. The system's pressure was then maintained at 1 bar using a Berendsenbarostat^49^. Every system was MD simulated for 20 ns.

In every simulation, the hydrogen bond atoms were constrained using the SHAKE approach. Every simulation integrated an SPFP precision model and used a 2 fs step size. Simulations were conducted using an isobaric-isothermal ensemble (NPT) with randomised seeding, constant pressure of 1 bar, pressure-coupling constant of 2 ps, temperature of 300 K, and Langevin thermostat with collision frequency of 1 ps.

#### Post-MD Analysis

The trajectories acquired via MD simulations were saved every 1 ps, and then they were examined using the CPPTRAJ module of the AMBER18 suite^50^. All graphs and visualizations were made using Chimera^38^ and the Origin data analysis tool^51^.

#### Thermodynamic calculation

Estimating ligand-binding affinities has been found to benefit from the Poisson-Boltzmann or generalized Born and surface area continuum solvation (MM/PBSA and MM/GBSA) approach^52,53^. Within a specified force field, the Protein–Ligand complex molecular simulations utilized by MM/GBSA and MM/PBSA provide accurate statistical-mechanical binding free energy. Average binding free energy over 200 images taken from the full 20 ns trajectory. According to Hou et al.^54^, the assessment of the change in binding free energy (ΔG) for each molecular species-complex, ligand, and receptor can be shown as follows:

1 $$ \Delta {\text{G}}_{{{\text{bind}}}} = {\text{G}}_{{{\text{complex}}}} - {\text{G}}_{{{\text{receptor}}}} - {\text{G}}_{{{\text{ligand}}}} $$

2 $$ \Delta {\text{G}}_{{{\text{bind}}}} = {\text{E}}_{{{\text{gas}}}} + {\text{G}}_{{{\text{sol}}}} - {\text{TS}} $$

3 $$ {\text{E}}_{{{\text{gas}}}} = {\text{E}}_{{{\text{int}}}} + {\text{E}}_{{{\text{vdw}}}} + {\text{E}}_{{{\text{ele}}}} $$

4 $$ {\text{G}}_{{{\text{sol}}}} = {\text{E}}_{{{\text{GB}}}} + {\text{G}}_{{{\text{SA}}}} $$

5 $$ {\text{G}}_{{{\text{SA}}}} = \gamma {\text{SASA}} $$

The gas-phase energy, internal energy, Coulomb energy, and van der Waals energy are represented by the terms Egas, Eint, Eele, and Evdw. The FF14SB force field words were used to directly assess the Egas. The energy involved from the polar states (GGB) and non-polar states (G) was used to calculate the solution-free energy (Gsol). Using a water probe radius of 1.4 Å, the non-polar solvation free energy (GSA) was calculated using the Solvent Accessible Surface Area (SASA)^55^. On the other hand, the polar solvation (GGB) contribution was evaluated by solving the GB equation. The total entropy of the solute and temperature are represented by items S and T, respectively.The total binding free energy of each residue was determined using the MM/GBSA-binding free energy method in Amber18.

#### Principal component analysis

PCA is a multivariate statistical method that screens observed motions from biggest to smallest spatial scale. It is used to systematically reduce the number of dimensions required to explain the dynamics of proteins. By identifying several conformational modes of the protein complex during dynamics simulations, PCA can be used to characterize the atomic displacement and conformational changes of proteins. PCA can also be used to ascertain the biological system's motion's direction (eigenvectors) and magnitude (eigen values). Here, the CPPTRAJ module in Amber18 was used to remove the solvent molecules and ions from 20 ns of MD trajectories^56^. This was completed before PCA's MD trajectory processing. Using proprietary scripts, PCA was carried out on Cα atoms for 1000 photos at 100 ps intervals. Using the Cartesian coordinates of the Cα atoms, the first two principal components (PC1 and PC2) were calculated and 2 × 2 covariance matrices were produced. The first two eigenvectors of a covariant matrix are represented as PC1 and PC2. Using Origin software, the PC plot was created.

#### DCCM analysis

Dynamic cross-correlation analysis was employed to examine the oscillations and motions within the α carbon atoms' backbone^57^. Based on structural information retrieved from MD trajectories, the cross-correlation elements Cij between Cα atoms of residues i and j of proteins can be estimated using the following equations^58^:

$$ {\text{C}}_{{{\text{ij}}}} = \frac{{ < \Delta {\text{ri}} \cdot \Delta {\text{rj}} > }}{{\left( { < \Delta {\text{r}}_{{\text{i}}}^{2} > < \Delta {\text{r}}_{{\text{j}}}^{2} > } \right)^{\frac{1}{2}} }} $$

In this case, Δri represents the ith Cα aom's displacement from its averaged position. The ith Cα atom's displacement from its averaged position is known as the Δri. Cij = 1 represents significantly correlated movements in the trajectory, whereas Cij = − -1 represents highly anticorrelated motions. The movements of i and j are anticorrelated, as seen by the divergence of motion from 1 and − 1. The CPPTRAJ package in Amber 18 was used to create the DCCM matrix, and Origin software (www.originlab.com) was used to plot and analyze the matrices^51^.

## Results and discussion

### Limonoids quantification

The demand for bioactive citrus limonoids has witnessed a significant surge due to their immense potential in enhancing health and their structure–activity relationship^59,60^. The challenge of scaling up techniques for isolating limonoids in sufficient quantities for specific biological studies has proven to be difficult. Therefore, it is crucial to explore alternative sources and methods for isolating citrus limonoids. Although there is a limited amount of information available regarding the yields of limonoids from *Citrus* limon peels, our study aims to contribute to this knowledge gap by providing a quantitative determination of limonoidaglycones and glycosides.

The concentration of limonoidaglycones in the extract of *C. limon* peels was determined using the colorimetric assay. The concentration was found to be 16.53 ± 0.93 mg/L limonin equivalent. This finding is consistent with a previous study by Pichaiyongvongdee and Haruenkit^61^, which reported that limoninaglycones ranged from 2 to 23 mg/L in different citrus fruits. Additionally, the total concentration of limonoidglucosides was measured to be 54.38 ± 1.02 mg/L. These values are similar to those documented in lemon juice (40–95 mg/L)^62^ and other *citrus* fruits^63^. The outcomes acquired from the assessment of limonoids concentration in *C. limon* peels reveal a notable disparity among different citrus fruits. This divergence is influenced by the collection place, agriculture techniques, and the fruit ripeness^64^. Additionally, this study successfully accomplished the isolation and determination of the chemical structure of five limonoids, namely limonin, deacetylnomilin, nomilin, obacunone 17-O-β-D-glucopyranoside, and limonin 17-O-β-D-glucopyranoside, along with their respective yields. These findings strongly support the notion that the peels of *C. limon* are a valid and valuable source of limonoids.

### Structure elucidation of the isolated limonoids

Compound 1, limonin, was isolated as white needles with a melting point of 119 °C. EI-MS revealed*m/z*470 for calculated molecular formula C_26_H_30_O_8_. The mass spectrum (Supplementary Fig. 1) also exhibited several characteristic peaks, including those at 413 (57%), 347 (100%), 329 (37%), 287(32%), 243 (29%), 225(34%), 187(40%), 147 (21%), 135 (38%), 108 (45%), 95(49%). ^1^H-NMR (CD_3_OD, 500 MHz) (Supplementary Fig. 2): δ ppm 4.11 (1H, s, H-1), 2.40 (1H, dd, J = 2.9, 15, H-2a), 2.58 (1H, dd, J = 1.7, 15.5 H-2b), 2.39 (1H, dd, J = 2.9, 13.5, H-5), 3.02 (1H, dd, J = 2.7, 15.5, H-6a), 2.99 (1H, t, J = 14.6, H-6b), 2.67 (1H, dd, J = 2.4, 11.7, H-9), 1.62 (1H, m, H-11a), 1.84 (1H, m, H-11b), 1.46 (1H, m, H12a), 1.59 (1H, m, H-12b), 4.23(1-H, s, H-15), 5.39 (1-H, s, H-17), 1.24 (3H, s, H-18), 4.53 (1H, d, J = 11.5, H19a), 4.60 (1H, d, J = 14.5, H-19b), 7.32 (1H, s, H-21), 6.11 (1H, s, H-22), 7.01 (1H, s, H-23),0.98 (3H, s, H-24), 1.12 (3H, s, H-25) and 1.15 (3H, s, H-26). ^13^C-NMR (100 MHz, CDCl3) (Supplementary Fig. 3): δ ppm 81.23 (C-1), 36.40 (C-2), 170.41 (C-3), 79.13 (C-4), 62.39 (C-5), 37.16 (C-6), 205.83 (C-7), 49.21 (C-8), 47.53 (C-9), 45.68 (C-10), 20.11 (C-11), 32.48 (C-12), 37.41 (C-13), 67.35 (C-14), 52.41 (C-15), 163.18 (C-16), 76.20 (C-17), 18.91 (C-18), 64.11 (C-19), 119.32 (C-20), 150.23 (C-21), 110.02 (C-22), 140.37 (C-23), 19.03 (C-24), 28.76 (C-25) and 20.46 (C-26). The results were concurred with the findings presented in previous studies conducted by Tian et al.^65^ and El-Sayed et al.^66^.

Compound 2, deacetylnomilin, was obtained as colorless needle-shaped crystals with a melting point of 254 °C. EI-MS (Supplementary Fig. 4) provided *m/z* 472 for molecular formula C_26_H_32_O_8_. Additionally, several characteristic peaks were observed at *m/z* 457 (61%), 315 (55%), 383 (40%), 349 (100%), 299 (48%), 243 (37%), 201 (25%), 135 (40%), 105 (38%), and 95 (67%). ^1^H-NMR (CD_3_OD, 500 MHz) (Supplementary Fig. 5): δ ppm 4.08 (1H, s, H-1),2.74 (1H, dd, J = 1.6,13.7 Hz, H-2a), 2.98 (1H, dd, J = 1.6, 14.5 Hz, H-2b), 2.38 (1H, dd, J = 2.3, 12.7 Hz, H-5), 2.76 (1H, dd, J = 14.3, 2.4 Hz, H-6a), 2.94 (1H, d, J = 14.3 Hz, H-6b), 2.81 (1H, dd, J = 1.8, 11.3 Hz, H-9), 1.54 (1H, m, H-11a),1.92 (1H, m, H-11b), 1.68 (1H, m, H-12a), 1.45 (1H, m, H-12b), 4.03 (1H, s, H-15), 5.27 (1H, s, H-17), 1.66 (3H, s, H-18), 1.32 (3H, s, H-19), 7.28 (1H, s, H-21), 6.24 (1H, s, H-22), 7.23 (1H, s, H-23), 0.87 (3H, s, H-24), 1.11 (3H, s, H-25), 1.04 (3H, s, H-26). ^13^C-NMR (100 MHz, CDCl3) (Supplementary Fig. 6): δ ppm 71.04 (C-1), 38.72 (C-2), 169.55 (C-3), 84.05 (C-4), 50.11 (C-5), 37.26 (C-6), 209.01 (C-7), 51.34 (C-8), 42.66 (C-9), 45.31 (C-10), 18.01 (C-11), 33.41 (C-12), 37.72 (C-13), 66.09 (C-14), 51.76 (C-15), 165.43 (C-16), 76.45 (C-17), 19.43 (C-18), 17.42 (C-19), 119.76 (C-20), 142.64 (C-21), 109.52 (C-22), 140.98 (C-23), 17.28 (C-24), 31.29 (C-25) and 22.58 (C-26). The outcomes were in accordance with the findings reported by Khalil et al.^67^.

Compound 3, nomilin, was acquired in the form of white crystals with a melting point of 278 ͦC. EI-MS (Supplementary Fig. 7) revealed *m/z* 514 for molecular formula C_28_H_34_O_9_. Furthermore, other distinctive peaks were observed at*m/z* 457 (61%), 315 (55%), 383 (40%), 349 (100%), 299 (48%), 243 (37%), 201 (25%), 135 (40%), 105 (38%), 95 (67%). ^1^H-NMR (CD_3_OD, 500 MHz) (Supplementary Fig. 8): δ ppm 4.11 (1H, s, H-1), 2.98 (1H, dd, J = 1.3, 12.5 Hz, H-2a), 2.64 (1H, dd, J = 1.3, 13.0 Hz, H-2b), 2.37 (1H, dd, J = 2.1, 12.9 Hz, H-5), 2.73 (1H, dd, J = 15.2, 2.4 Hz, H-6a), 2.78 (1H, d, J = 15.2 Hz, H-6b), 1.61 (1H, m, H-11a), 1.63 (1H, m, H-11b), 1.70 (1H, m, H-12a), 1.72 (1H, m, H-12b),4.12 (1H, s, H-15), 5.03 (1H, s, H-17), 1.57 (3H, s, H-18), 1.28 (3H, s, H-19), 7.19 (1H, s, H-21), 6.13 (1H, s, H-22), 6.45 (1H, s, H-23), 0.96 (3H, s, H-24), 1.24 (3H, s, H-25), 1.36 (3H, s, H-26). ^13^C-NMR (100 MHz, CDCl3) (Supplementary Fig. 9): δ ppm 69.86 (C-1), 36.44 (C-2), 167.43 (C-3), 80.03 (C-4), 50.43 (C-5), 37.64 (C-6), 207.08 (C-7), 51.67 (C-8), 43.91 (C-9), 45.02 (C-10), 17.84 (C-11), 33.08 (C-12), 36.96 (C-13), 66.13 (C-14), 52.82 (C-15), 165.89 (C-16), 77.63 (C-17), 18.06 (C-18), 18.00 (C-19), 119.85 (C-20), 142.87 (C-21), 108.49 (C-22), 140.65 (C-23), 24.42 (C-24), 21.47 (C-25), 21.58 (C-26), 35.16 (CH_3_-Acetyl)and 170.31 (CO). The data aligns with the findings put forth by Mandadi et al.^18^.

Compound 4, obacunone 17-O-β-D-glucopyranoside, was obtained ascolorless amorphous solid with a melting point of 215ͦ C. EI-MS (Supplementary Fig. 10) demonstrated the presence of a molecular ion at *m/z* 634, which corresponds to the calculated molecular formula of C_32_H_42_O_13_. The characteristic fragments identified as 560 (25%), 472 (65%), 426 (100%), 398 (48%), 260 (23%), and 97 (61%). ^1^H-NMR (CD_3_OD, 500 MHz) (Supplementary Fig. 11): δ ppm 6.34 (1H, d, J = 11.3 Hz, H-1), 6.12 (1-H, d, J = 11.3 Hz, H-2), 3.05 (1H, s, H-15), 1.67 (3H, s, H-18), 7.03 (1H, d, J = 7.4 Hz, H-21), 6.87 (1H, s, H-22), 7.52 (1H, d, J = 7.4 Hz, H-23), 1.52 (3H, s, H-19), 1.39 (3H, s, H-24), 1.08 (3H, s, H-25), 0.96 (3H, s, H-26), 4.85 (1H, d, = 9.2 Hz, anomericproton), sugar protons at 3.27–1.54 ppm. ^13^C-NMR (CD_3_OD, 125 MHz) (Supplementary Fig. 12): δ ppm 153.6 (C-1), 120.1 (C-2), 170.3 (C-3), 84.7 (C-4), 52.6 (C-5), 41.8 (C-6), 211.4 (C-7), 52.7 (C-8), 49.8 (C-9), 45.7 (C-10), 21.3 (C-11), 29.4 (C-12), 45.8 (C-13), 69.8 (C-14), 58.6 (C-15), 169.7 (C-16), 79.4 (C-17), 126.5 (C-20), 144.2 (C-21), 114.6 (C-22), 142.5 (C-23), 16.2(CH_3_), 20.4(CH_3_), 22.3(CH_3_), 25.4 (CH_3_), 28.7(CH_3_), 19.03 (CO),104.8 (C-1'), 74.6(C-2'), 74.8 (C-3'), 71.3 (C-4'), 69.0 (C-5'), 61.5 (C-6'). The data is in agreement with the findings outlined by Sawabe et al.^68^.

**Fig. 1:**
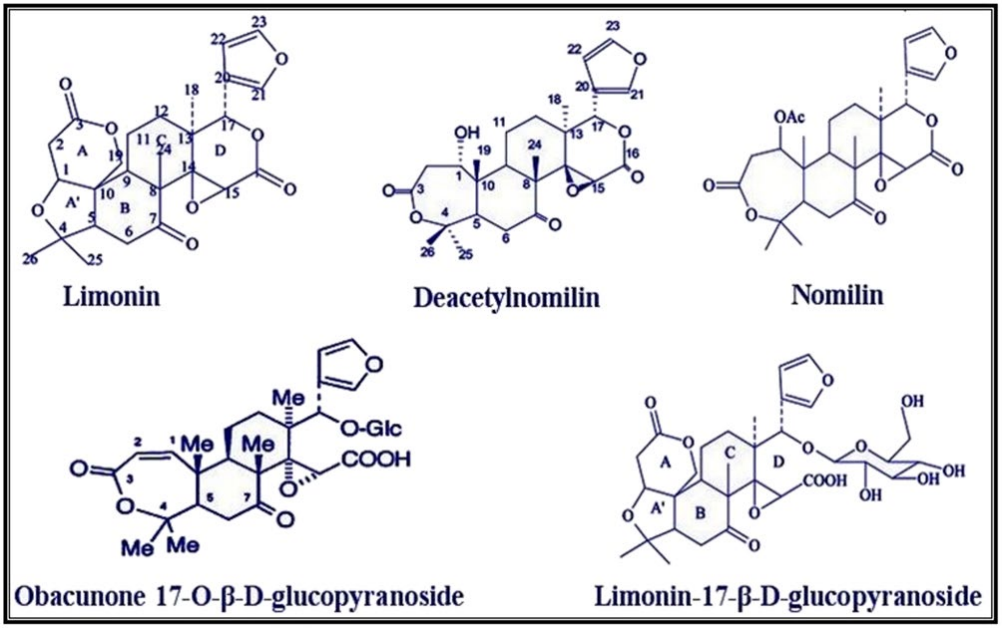
The structure of the isolated limonoids.

Compound 5, limonin 17-O-β-D-glucopyranoside, was isolated as yellow crystals witha melting point of 235 ͦ C. EI-MS (Supplementary Fig. 13) demonstrated the presence of a molecular ion at *m/z* 650, which corresponds to the calculated molecular formula of C_32_H_42_O_14_.The characteristic fragments identified as 489 (51%), 471 (46%), 403 (31%), 247 (24%), 125 (100%), and 97 (73%). The structure of the compound was further elucidated through the analysis of its ^1^H-NMR and ^13^C-NMR data. ^1^H-NMR (CD_3_OD, 500 MHz) (Supplementary Fig. 14): δ ppm 4.25 (1H, s, H-1), 4.91 (1-H, d, J = 9.8 Hz, H-15), 5.21 (1H, s, H-17), 1.29 (3H, s, H-19), 6.98 (1H, d, J = 6.8 Hz, H-21), 6.73 (1H, s, H-22), 7.34 (1H, d, J = 6.8 Hz, H-23), 0.87 (3H, s, H-24), 1.46 (3H, s, H-25), 0.72 (3H, s, H-26), 4.36 (1H, d, anomeric proton), sugar protons at 3.35- 1.61 ppm. ^13^C-NMR (CD_3_OD, 125 MHz) (Supplementary Fig. 15): δ ppm 79.6 (C-1), 36.4 (C-2), 169.8 (C-3), 78.2 (C-4), 56.4 (C-5), 37.5 (C-6), 207.5 (C-7), 51.4 (C-8), 44.6 (C-9), 46.3 (C-10), 18.4 (C-11), 27.5 (C-12), 43.7 (C-13), 69.5 (C-14), 55.8 (C-15), 169.3 (C-16), 76.8 (C-17), 24.1 (C-18), 125.7 (C-20), 140.3 (C-21), 112.5 (C-22), 139.8 (C-23), 31.7(CH_3_), 24.1(CH_3_), 21.4(CH_3_), 16.7 (CH_3_), 18.2 (CO),103.7 (C-1'), 74.2 (C-2'), 73.2 (C-3'), 72.0 (C-4'), 68.2 (C-5'), 59.3 (C-6'). The identification of the compound was corroborated by the results presented in the study conducted by Mandadi et al.^18^. This specific compound was found to be the most abundant glucoside limonoid detected in lemons, limes, oranges, and grapefruits^69,70^. It, along with limonin and nomilin, has been identified as the cause of the bitter taste in *citrus* fruits and contributes to the overall flavor profile and health benefits of these fruits, as mentioned by Samanta et al.^71^. The structure of the isolated limonoids is illustrated in Fig. 1. Structure–activity relationship of the limonoids is characterized by the presence of five rings, namely A, A', B, C, and D. Deacetylnomilin and nomilin differ from limonin in that they lack the A' ring, while they vary from each other in the substitution at C-1. Furthermore, upon the maturation of citrus fruits, glucosidation is triggered in the D ring, as evidenced by B'chir and Arnaud^72^, resulting in the isolation of limonin 17-O-β-D-glucopyranoside and obacunone 17-O-β-D-glucopyranoside. From a biological standpoint, previous studies have provided evidence that the presence of the intact A ring and a furan moiety in the limonoid skeleton enhances its potential^73^.

### In vitro biological activities

#### Antioxidant activity

The active phyto-constituents exhibit their antioxidant action by breaking the free radical chain through the donation of a hydrogen atom^74^. The IRP assay is reproducible and linearly related to the antioxidant activity. During this assay, the antioxidant acts as electron donor to reduce the iron from ferric (Fe^3+^) to its ferrous (Fe^2+^) state and the reducing power is indicated by higher absorbance values^75^. During the present study, the antioxidant activity of the limonoids-rich extract and the five isolated compounds was assessed at three concentrations (50, 100, and 200 µg/mL) by measuring TAC and IRP. It was found that both TAC and IRP increased as the concentration of the samples increased, indicating a concentration-dependent relationship **(**Table 1**)**. Specifically, the samples at a concentration of 200 µg/mL exhibited the highest antioxidant activity compared to the other lower concentrations (50 and 100 µg/mL). In comparison to the other samples, both obacunone 17-O-β-D-glucopyranoside (Cpd4) and limonin 17-O-β-D-glucopyranoside (Cpd5) possessed the highest antioxidant activity, followed by deacetylnomilin, and then the limonoids-rich extract. At the highest concentration studied (200 µg/mL), both obacunone 17-O-β-D-glucopyranoside (Cpd4) and limonin 17-O-β-D-glucopyranoside (Cpd5) exhibited higher TAC (86.10 ± 0.23 and 86.96 ± 0.23 mg gallic acid/g, respectively) and IRP (69.78 ± 0.23 and 70.65 ± 0.23 µg/mL, respectively). This is in agreement with Sánchez-Marzo et al.^76^, who demonstrated that the presence of higher functional groups, including hydroxyl and carboxyl groups, as well as a methyl group, was responsible for the strongest antioxidant and reducing power compared to the other isolated compounds. In contrast, at the same concentration, the TAC and IRP of standard ascorbic acid were 95.34 ± 0.25 mg gallic acid/g and 75.90 ± 0.23 µg/mL, respectively.

**Table 1:** The antioxidant activity of limonoids-rich extract and the isolated compounds at 3 different concentrations.

Sample	Concentration (µg/mL)	TAC (mg gallic acid/g)	IRP (µg/mL)
Ext	50	18.66 ± 0.03	13.51 ± 0.03
	100	33.22 ± 0.05	24.05 ± 0.05
	200	59.13 ± 0.09	42.82 ± 0.09
Cpd1	50	12.77 ± 0.03	7.62 ± 0.03
	100	22.73 ± 0.04	13.56 ± 0.04
	200	40.46 ± 0.08	24.14 ± 0.08
Cpd2	50	21.74 ± 0.06	16.59 ± 0.06
	100	38.70 ± 0.10	29.53 ± 0.10
	200	68.88 ± 0.19	52.56 ± 0.19
Cpd3	50	12.90 ± 0.03	7.75 ± 0.03
	100	22.96 ± 0.05	13.79 ± 0.05
	200	40.87 ± 0.08	24.55 ± 0.08
Cpd4	50	27.18 ± 0.07	22.03 ± 0.07
	100	48.37 ± 0.13	39.20 ± 0.13
	200	86.10 ± 0.23	69.78 ± 0.23
Cpd5	50	27.45 ± 0.07	22.30 ± 0.07
	100	48.86 ± 0.13	39.69 ± 0.13
	200	86.96 ± 0.23	70.65 ± 0.23
Ascorbic Acid	50	30.09 ± 0.08	23.95 ± 0.07
	100	53.56 ± 0.14	42.64 ± 0.13
	200	95.34 ± 0.25	75.90 ± 0.23

Values are given as mean ± standard error (calculated from three replicates), Orange cell indicates the most active sample. **Ext.**: Limonoids-rich extract, **Cpd1**: Limonin, **Cpd2**: Deacetylnomilin, **Cpd3**: Nomilin, **Cpd4**: Obacunone 17-O-β-D-glucopyranoside, **Cpd5**: Limonin 17-O-β-D-glucopyranoside.

The lowest TAC and IRP were noticed with limonin (Cpd1) (40.46 ± 0.08 mg gallic acid/g and 24.14 ± 0.08 µg/mL, respectively) and nomilin (Cpd3) (40.87 ± 0.08 mg gallic acid/g and 24.55 ± 0.08 µg/mL, respectively). This is in accordance with Breksa and Manners^77^ and supported consequently by Zou et al.^78^ who postulated that both limonin and nomilin showed antioxidant effect equivalent to cinnamic acid used as a negative control, indicating that they do not possess an inherent antioxidant capacity.

#### Scavenging activity

It was assessed by calculating the inhibition percentages at three different concentrations (50, 100, and 200 µg/mL) and the median inhibitory concentrations (IC_50_) of the limonoids-rich extract and the five isolated compounds against DPPH, ABTS, and NO radicals. Data presented in Table 2 showed that the scavenging activity against these radicals increased in all studied samples as their concentrations increased. At a concentration of 200 µg/mL, each sample possessed higher scavenging activity compared to the other concentrations (50 and 100 µg/mL). This followed data of their antioxidant activity (TAC and IRP). It was found that both obacunone 17-O-β-D-glucopyranoside (Cpd4) and limonin 17-O-β-D-glucopyranoside (Cpd5) exhibited the highest inhibitory activity against DPPH (74.87 ± 0.20 and 75.62 ± 0.20%, respectively), ABTS (69.78 ± 0.23 and 70.65 ± 0.23%, respectively) and NO radicals (58.55 ± 0.20 and 59.30 ± 0.20%, respectively), followed by deacetylnomilin (Cpd2) (59.90 ± 0.16, 52.56 ± 0.19 and 43.58 ± 0.16%, respectively), and then the limonoids-rich extract (Ext.) (51.42 ± 0.08, 42.82 ± 0.09 and 35.10 ± 0.08%, respectively). This might be related to the high molecular weight, the number of aromatic rings, and the nature of hydroxyl group substitutions, which increase the ability to quench free radicals more than specific functional groups^74^. Furthermore, both obacunone 17-O-β-D-glucopyranoside and limonin 17-O-β-D-glucopyranoside exhibited the highest scavenging activity due to the participation of the hydroxyl groups, which are characterized by their ability to donate an electron or hydrogen ion, which may then interact with the reactive species (accept an electron or hydrogen radical) to become a stable diamagnetic molecule^79,80^.

**Table 2:** The scavenging activity of limonoids-rich extract and the isolated compounds at 3 different concentrations.

Sample	Concentration (µg/mL)	DPPH	ABTS	NO
		Inhibition (%)		
Ext	50	16.23 ± 0.03	13.51 ± 0.03	11.08 ± 0.03
	100	28.89 ± 0.04	24.05 ± 0.05	19.72 ± 0.04
	200	51.42 ± 0.08	42.82 ± 0.09	35.10 ± 0.08
Cpd1	50	11.10 ± 0.02	7.62 ± 0.03	5.95 ± 0.02
	100	19.77 ± 0.04	13.56 ± 0.04	10.60 ± 0.04
	200	35.18 ± 0.07	24.14 ± 0.08	18.87 ± 0.07
Cpd2	50	18.90 ± 0.05	16.59 ± 0.06	13.75 ± 0.05
	100	33.65 ± 0.09	29.53 ± 0.10	24.48 ± 0.09
	200	59.90 ± 0.16	52.56 ± 0.19	43.58 ± 0.16
Cpd3	50	11.22 ± 0.02	7.75 ± 0.03	6.07 ± 0.02
	100	19.96 ± 0.04	13.79 ± 0.05	10.80 ± 0.04
	200	35.53 ± 0.07	24.55 ± 0.08	19.22 ± 0.07
Cpd4	50	23.63 ± 0.06	22.03 ± 0.07	18.48 ± 0.06
	100	42.06 ± 0.11	39.20 ± 0.13	32.90 ± 0.11
	200	74.87 ± 0.20	69.78 ± 0.23	58.55 ± 0.20
Cpd5	50	23.87 ± 0.06	22.30 ± 0.07	18.72 ± 0.06
	100	42.48 ± 0.11	39.69 ± 0.13	33.32 ± 0.11
	200	75.62 ± 0.20	70.65 ± 0.23	59.30 ± 0.20
Ascorbic Acid	50	26.17 ± 0.07	24.94 ± 0.08	21.02 ± 0.07
	100	45.43 ± 0.11	42.64 ± 0.13	36.27 ± 0.11
	200	80.87 ± 0.20	75.90 ± 0.23	64.55 ± 0.20

Values are given as mean ± standard error (calculated from three replicates), Orange cell indicates the most active sample. **Ext.**: Limonoids-rich extract, **Cpd1**: Limonin, **Cpd2**: Deacetylnomilin, **Cpd3**: Nomilin, **Cpd4**: Obacunone 17-O-β-D-glucopyranoside, **Cpd5**: Limonin 17-O-β-D-glucopyranoside.

The lowest scavenging activity was noticed with limonin (Cpd1) (35.18 ± 0.07, 24.14 ± 0.08 and 18.87 ± 0.07%, respectively) and nomilin (Cpd3) (35.53 ± 0.07, 24.55 ± 0.08 and 19.22 ± 0.07%, respectively). This agrees with Yu et al.^81^ who emphasize that both limonin and nomilin have reduced radical scavenging capacity, although the juices demonstrated a higher capacity than the extracts from fruit tissues due to a decrease in their antioxidant properties, which is related to the fewer hydroxyl and carboxyl groups.

**Fig. 2:**
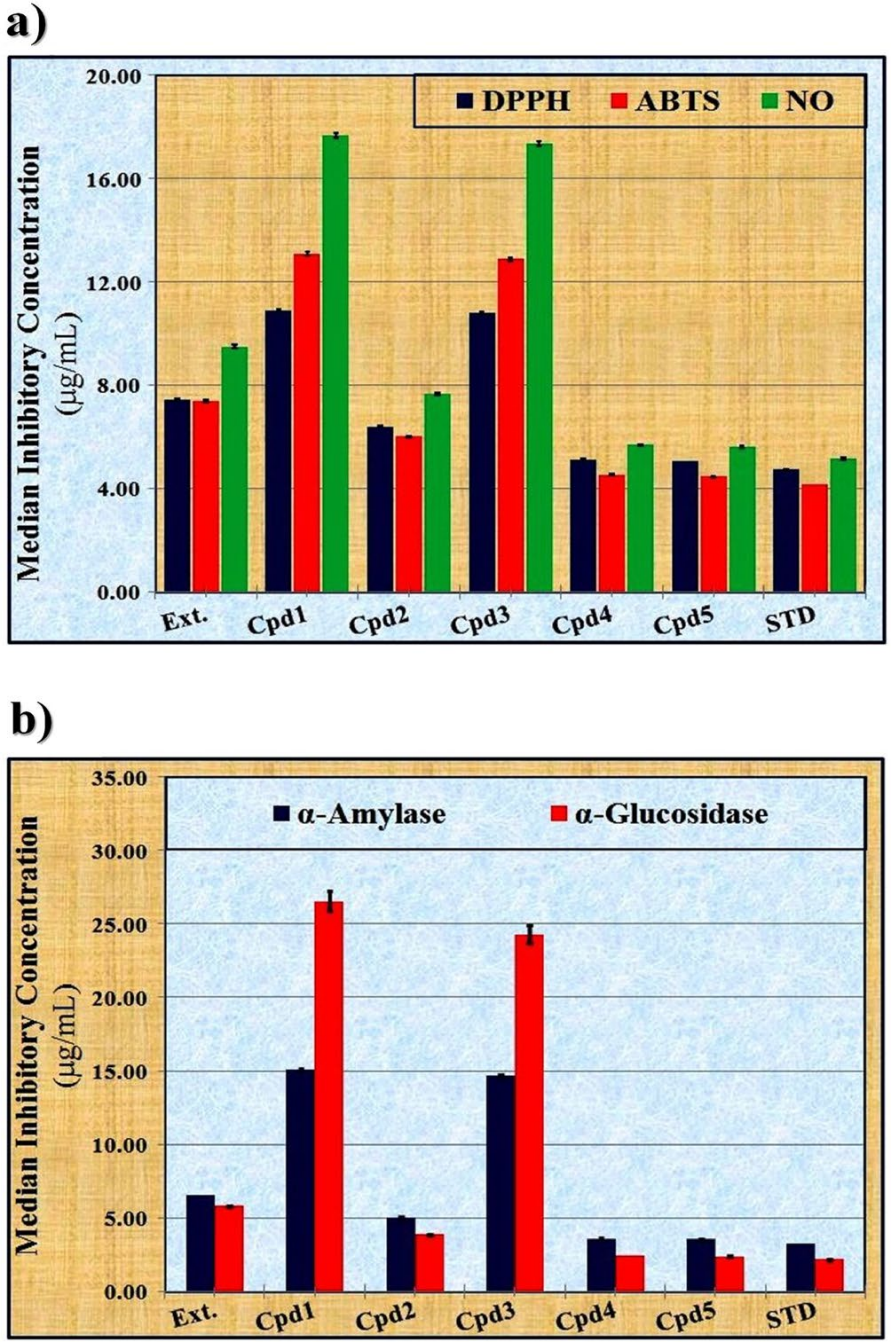
The median inhibitory concentrations (IC_50_) of limonoids-rich extract and the isolated compounds against (**a**) free radicals (DPPH, ABTS and NO), (**b**) hydrolases enzymes (α-amylase and α-glucosidase). Values are given as mean ± standard error (calculated from three replicates). **Ext.**: Limonoids-rich extract, **Cpd1**: Limonin, **Cpd2**: Deacetylnomilin, **Cpd3**: Nomilin, **Cpd4**: Obacunone 17-O-β-D-glucopyranoside, **Cpd5**: Limonin 17-O-β-D-glucopyranoside.

At the same concentration, ascorbic acid (the standard) inhibited the DPPH, ABTS, and NO radicals by 80.87 ± 0.20, 75.90 ± 0.23 and 64.55 ± 0.20%, respectively. The data presented in Fig. 2a show values of the IC_50_ against DPPH, ABTS, and NO radicals. The inhibition percentages (%) are inversely proportional to the values of the IC_50_. The lowest IC_50_ value indicates the highest inhibitory effect against the free radicals and hence the highest scavenging activity.

From this point, the lowest IC_50_ values against DPPH, ABTS, and NO radicals were observed with both obacunone 17-O-β-D-glucopyranoside (Cpd4) (5.12 ± 0.02, 4.53 ± 0.02 and 5.70 ± 0.03 µg/mL, respectively) and limonin 17-O-β-D-glucopyranoside (Cpd5) (5.07 ± 0.02, 4.48 ± 0.02 and 5.62 ± 0.03 µg/mL, respectively), followed by deacetylnomilin (Cpd2) (6.40 ± 0.02, 6.02 ± 0.03 and 7.65 ± 0.04 µg/mL, respectively), and then the limonoids-rich extract (Ext.) (7.46 ± 0.03, 7.39 ± 0.04 and 9.50 ± 0.06 µg/mL, respectively). The IC_50_ values calculated for ascorbic acid were 4.74 ± 0.02, 4.17 ± 0.02, and 5.17 ± 0.03 µg/mL, respectively.

#### The anti-diabetic activity

The α-amylase is categorized as a digestive enzyme that catalyzes the hydrolysis of dietary carbohydrates into oligosaccharides and disaccharides by cleaving the α-(1,4)-D-glycosidic linkages^82^. The α-glucosidase is a crucial enzyme in carbohydrate digestion and causes postprandial hyperglycemia by stimulating the release of glucose as a result of breaking down linear and branched oligosaccharides^83^. The therapeutic strategy required for controlling diabetes mellitus is to retard the absorption of glucose by inhibiting these hydrolytic enzymes in the digestive organs^84^. During the current study, the anti-diabetic activity was determined by calculating the inhibition percentages at three different concentrations (50, 100, and 200 µg/mL) and the IC_50_ of the limonoids-rich extract and the five isolated compounds against the activity of α-amylase and α-glucosidase enzymes. The data depicted in Table 3 show that the anti-diabetic activity follows the pattern of the antioxidant and scavenging activities, which increased in all studied samples with increasing concentrations. Therefore, the sample that exhibited higher antioxidant and scavenging activities at a concentration of 200 µg/mL also showed higher anti-diabetic activity compared to the other concentrations (50 and 100 µg/mL). This agrees with Sarian et al.^85^, who suggested that the total number and configuration of hydroxyl groups are responsible for regulating the antioxidant and anti-diabetic properties of the active constituents, thereby improving the activities of both α-amylase and α-glucosidase. Moreover, the substitution of hydroxyl groups with methoxy groups, in addition to the presence of double bonds, is able to increase the anti-diabetic activity^86,87^. In addition, these phyto-constituents inhibit the activity of these enzymes by interacting with the enzyme through non-specific binding. Their inhibitory activity increases with molecular weight and degree of polymerization^88^. Therefore, both obacunone 17-O-β-D-glucopyranoside (Cpd4) and limonin 17-O-β-D-glucopyranoside (Cpd5) exhibited the highest inhibition percentages against the activity of α-amylase (59.96 ± 0.23 and 60.82 ± 0.23%, respectively) and α-glucosidase enzyme (46.81 ± 0.23 and 47.67 ± 0.23, respectively), followed by deacetylnomilin (Cpd2) (42.74 ± 0.19 and 29.59 ± 0.19%, respectively), and then the limonoids-rich extract (Ext.) (32.99 ± 0.09 and 19.84 ± 0.09%, respectively). At the same concentration, Acarbose (the standard) inhibited the activity of both α-amylase and α-glucosidase enzymes by 66.07 ± 0.23 and 52.92 ± 0.23%, respectively.

**Table 3:** The in vitro anti-diabetic activity of limonoids-rich extract and the isolated compounds at 3 different concentrations.

Sample	Concentration (µg/mL)	α-Amylase	α-Glucosidase
		Inhibition (%)	
Ext	50	10.41 ± 0.03	6.26 ± 0.03
	100	18.54 ± 0.05	11.15 ± 0.05
	200	32.99 ± 0.09	19.84 ± 0.09
Cpd1	50	4.52 ± 0.03	1.37 ± 0.03
	100	8.05 ± 0.04	2.44 ± 0.04
	200	14.32 ± 0.08	4.34 ± 0.08
Cpd2	50	13.49 ± 0.06	9.34 ± 0.06
	100	24.01 ± 0.10	16.63 ± 0.10
	200	42.74 ± 0.19	29.59 ± 0.19
Cpd3	50	4.65 ± 0.03	1.50 ± 0.03
	100	8.27 ± 0.05	2.67 ± 0.05
	200	14.73 ± 0.08	4.75 ± 0.08
Cpd4	50	18.93 ± 0.07	14.78 ± 0.07
	100	33.69 ± 0.13	26.30 ± 0.13
	200	59.96 ± 0.23	46.81 ± 0.23
Cpd5	50	19.20 ± 0.07	15.05 ± 0.07
	100	34.17 ± 0.13	26.78 ± 0.13
	200	60.82 ± 0.23	47.67 ± 0.23
Acarbose	50	21.84 ± 0.08	17.69 ± 0.08
	100	37.12 ± 0.13	29.73 ± 0.13
	200	66.07 ± 0.23	52.92 ± 0.23

Values are given as mean ± standard error (calculated from three replicates), Orange cell indicates the most active sample. **Ext.**: Limonoids-rich extract, **Cpd1**: Limonin, **Cpd2**: Deacetylnomilin, **Cpd3**: Nomilin, **Cpd4**: Obacunone 17-O-β-D-glucopyranoside, **Cpd5**: Limonin 17-O-β-D-glucopyranoside.

As depicted in Table 4, it was emphasized that antioxidant activity (TAC and IRP), the scavenging activity against DPPH, ABTS, and NO radicals, and anti-diabetic activity are positively (at *p* ≤ 0.01) correlated with each other at equal concentrations of the limonoids-rich extract and the isolated compounds. Therefore, compounds with high antioxidant activity exhibit a strong capacity to inhibit the activity of carbohydrate digestive enzymes (α-amylase and α-glucosidase)^89^. From this point of view, the lowest anti-diabetic activity was noticed with limonin (Cpd1) (14.32 ± 0.08 and 4.34 ± 0.08%, respectively) and nomilin (Cpd3) (14.73 ± 0.08 and 4.75 ± 0.08%, respectively). This can be attributed to their lower antioxidant and scavenging activities compared to the other isolated compounds, which exhibit different levels of antioxidant activity depending on their structure and chemical function^90^. The lowest IC_50_ value indicates the highest inhibitory effect against activity of both α-amylase and α-glucosidase enzymes and hence the highest anti-diabetic activity. The lowest IC_50_ values were observed with both obacunone 17-O-β-D-glucopyranoside (Cpd4) (3.61 ± 0.02 and 2.46 ± 0.02 µg/mL, respectively) and limonin 17-O-β-D-glucopyranoside (Cpd5) (3.56 ± 0.02 and 2.42 ± 0.02 µg/mL, respectively), followed by deacetylnomilin (5.07 ± 0.03 and 3.89 ± 0.03 µg/mL, respectively), and then the limonoids-rich extract (Ext.) (6.57 ± 0.01 and 5.81 ± 0.07 µg/mL, respectively). The IC_50_ values calculated for Acarbose were 3.28 ± 0.02, and 2.18 ± 0.02 µg/mL, respectively **(**Fig. 2b**)**.

**Table 4:** The statistical correlations among the different in vitro biological activities of limonoids-rich extract and the isolated compounds at equal concentrations (100 µg/mL).

		Antioxidant activity		Scavenging activity			Anti-diabetic activity	
		TAC	IRP	DPPH	ABTS	NO	α-Amylase	α-Glucosidase
Antioxidant	TAC	–	0.000**	0.000**	0.000**	0.000**	0.000**	0.000**
	IRP	0.000**	–	0.000**	0.000**	0.000**	0.000**	0.000**
Scavenging	DPPH	0.000**	0.000**	–	0.000**	0.000**	0.000**	0.000**
	ABTS	0.000**	0.000**	0.000**	–	0.000**	0.000**	0.000**
	NO	0.000**	0.000**	0.000**	0.000**	–	0.000**	0.000**
Anti-diabetic	α-Amylase	0.000**	0.000**	0.000**	0.000**	0.000**	–	0.000**
	α-Glucosidase	0.000**	0.000**	0.000**	0.000**	0.000**	0.000**	–

** The correlation is significant at *p* ≤ 0.01.

The different proteins and isoenzymes were separated, identified, and quantified by electrophoresis, which is well known for its ability to analyze the stoichiometry of a specific subunit of a protein complex. Moreover, it can be used to reveal qualitative differences by either hiding normal bands and/or showing the appearance of abnormal ones^91^. The physiological state of the protein and enzyme can be assessed by the percentage of the SI%^92^. When the number and arrangement of electrophoretically separated bands differed, the SI% values decreased compared to the control group indicating qualitative alterations. The SI% values are not linked to quantitative changes^93^.

**Fig. 3:**
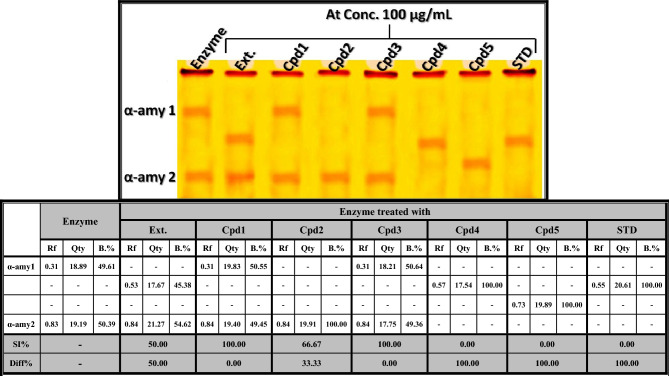
Native electrophoretic α-amylase isoenzymes pattern showing the anti-diabetic activity of limonoids-rich extract and the isolated compounds compared to Acarbose (standard) on the physiological state of α-amylase enzyme. **Ext.**: Limonoids-rich extract, **Cpd1**: Limonin, **Cpd2**: Deacetylnomilin, **Cpd3**: Nomilin, **Cpd4**: Obacunone 17-O-β-D-glucopyranoside, **Cpd5**: Limonin 17-O-β-D-glucopyranoside.

As shown in Fig. 3, it was observed that the standard α-amylase enzyme was electrophoretically represented by two types identified at Rfs 0.31 and 0.83 (Qty 18.89 and 19.19; B% 49.61 and 50.39, respectively). The standard enzyme was treated with an equal concentration (100 µg/mL) of limonoids-rich extract and the five isolated compounds. Treatment of the standard (α-amylase) enzyme with both limonin and nomilin caused no qualitative or quantitative variations in the electrophoretic isoenzyme pattern. Therefore, the electrophoretic α-amylase pattern in these samples is physiologically similar to the standard enzyme (SI = 100.00%; Diff. = 0.00%). Treatment of the standard enzyme with the limonoids-rich extract (Ext.) caused changes in the electrophoretic isoenzyme pattern, indicated by the disappearance of one normal type (α-amy 1) and the presence of one abnormal band identified at Rf 0.53 (Qty 17.67 and B.% 45.38). Therefore, the electrophoretic α-amylase pattern with this sample is physiologically similar to the standard enzyme by 50.00% (SI = 50.00%; Diff. = 50.00%). Treatment of the standard enzyme with deacetylnomilin (Cpd2) caused slight changes in the electrophoretic isoenzyme pattern, represented by hiding one normal type (α-amy 1) without the appearance of abnormal bands. Therefore, the electrophoretic α-amylase pattern of this sample is physiologically similar to the standard enzyme by 66.67% (SI = 66.67%; Diff. = 33.33%). Regarding both obacunone 17-O-β-D-glucopyranoside and limonin 17-O-β-D-glucopyranoside, it was found that they caused severe abnormalities, as indicated by the absence of normal α-amylase bands and the presence of one abnormal band at Rf 0.57 (Qty 17.54 and B.% 100.00) with obacunone 17-O-β-D-glucopyranoside (Cpd4), and at Rf 0.73 (Qty 19.89 and B.% 100.00) with limonin 17-O-β-D-glucopyranoside (Cpd5). Therefore, the electrophoretic α-amylase pattern of these samples is completely different from the standard enzyme in its physiological state (SI = 0.00%; Diff. = 100.00%). As demonstrated by Aboulthana et al.^94^, the alterations in the electrophoretic α-amylase isoenzyme pattern might be attributed to the fractional and structural changes induced in the protein portion of native enzymes, which consequently lead to changing the enzymatic activities of the α-amylase isoenzyme pattern. The standard drug (Acarbose) caused complete denaturation in the electrophoretic isoenzyme pattern, as represented electrophoretically by obscuring all the isoenzyme types without the appearance of abnormal bands.

**Fig. 4:**
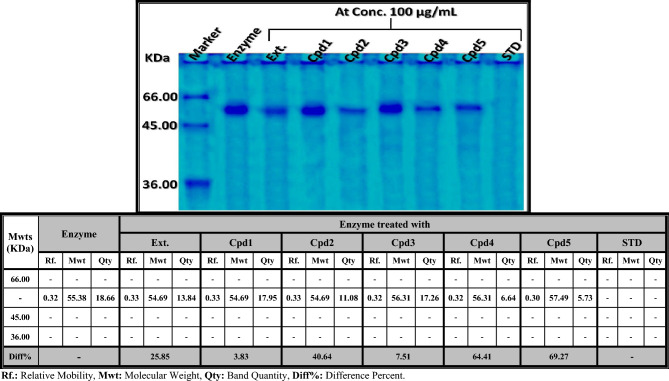
Native electrophoretic protein pattern showing the anti-diabetic activity of limonoids-rich extract and the isolated compounds on the quantity of the α-glucosidase enzyme checked by SDS PAGE compared to Acarbose (standard). **Ext.**: Limonoids-rich extract, **Cpd1**: Limonin, **Cpd2**: Deacetylnomilin, **Cpd3**: Nomilin, **Cpd4**: Obacunone 17-O-β-D-glucopyranoside, **Cpd5**: Limonin 17-O-β-D-glucopyranoside.

As shown in Fig. 4, the α-glucosidase was visualized and confirmed using SDS-PAGE as a single band with a molecular weight of 43 kDa, which agrees with the experiment carried out by El-Shora et al.^95^. During the current study, the crude α-glucosidase enzyme was identified as a single band at Rf 0.32 (Qty 55.38 and B% 18.66). SDS-PAGE was used to reveal the quantitative differences induced by the limonoids-rich extract and the five isolated compounds at equal concentrations (100 µg/ mL) and detected electrophoretically. It was found that both obacunone 17-O-β-D-glucopyranoside (Cpd4) and limonin 17-O-β-D-glucopyranoside (Cpd5) exhibited the highest denaturing activity against α-glucosidase, represented by a decrease in the quantity of the identified band by 64.41% (Qty 6.64) and 69.27% (Qty 5.73), respectively. This was followed by deacetylnomilin (Cpd2), which decreased the quantity of the band by 40.64% (Qty 11.08), and then the limonoids-rich extract (Ext.), which decreased the quantity of the identified band by 25.85% (Qty 13.84). The altered electrophoretic α-glucosidase pattern might be potentially attributable to the ionization of certain amino acid side chains and the formation of side-products, which cause denaturation of the α-glucosidase protein and hence inhibit enzyme activity^96^. The lowest anti-diabetic activity was observed with both limonin and nomilin, which slightly decreased the quantity of the identified band by 3.83% (Qty 17.95) and 7.51% (Qty 17.26), respectively. The standard drug (Acarbose) caused complete denaturation in the electrophoretic isoenzyme pattern, as represented electrophoretically by obscuring the identified band completely.

### Molecular docking and molecular dynamic (MD) simulations

#### Molecular dynamic and system stability

**Fig. 5:**
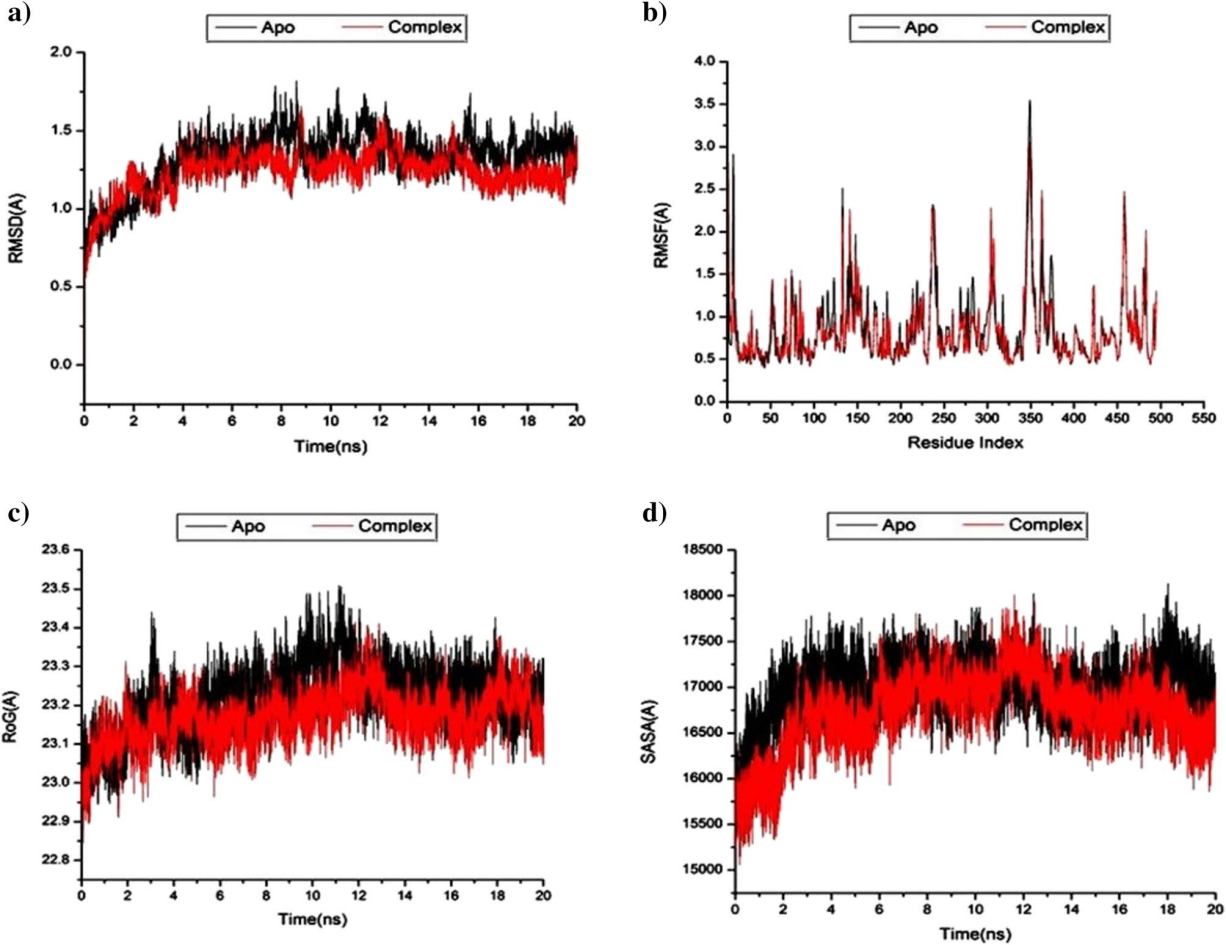
**a)** RMSD of Cα atoms of the protein backbone atoms, **b)** RMSF of each residue of the protein backbone α atoms of protein residues, **c)** ROG of Cα atoms of protein residues and **d)** solvent accessible surface area (SASA) of the C α of the backbone atoms relative (black) to the starting minimized over 20 ns for the ATP binding site of HPA receptor with Limonin 17-O-β-D-glucopyranoside (red).

To forecast how the separated compounds would behave upon binding to the protein's active region as well as how they would interact and remain stable, a molecular dynamic simulation was run^97,98^. To identify interrupted motions and prevent any artifacts during the simulation, system stability must be validated. The stability of the systems was evaluated in this study using Root-Mean-Square Deviation (RMSD) during the 20 ns simulations. For the complete frames of the systems, the average RMSD values were 1.33 ± 0.17 Å for HPA -apo and 1.23 ± 0.13 Å for HPA—limonin 17-O-β-D-glucopyranoside complex **(**Fig. 5a**)**. These findings showed that compared to the Apo system, the limonin 17-O-β-D-glucopyranoside -bound to protein complex system developed a comparatively more stable shape.

Analyzing the structural flexibility of proteins following ligand binding during MD simulation is essential for investigating residue behavior and their relationship to the ligand^99^. Using the Root-Mean-Square Fluctuation (RMSF) technique, protein residue variations were assessed to determine the impact of inhibitor binding to the corresponding targets during 20 ns simulations. The RMSF values that were estimated for HPA -apo and HPA—limonin 17-O-β-D-glucopyranoside complex were 0.85 ± 0.43 Å and 0.81 ± 0.41 Å, respectively **(**Fig. 5b**)**. These results showed that, in comparison to the other systems, the limonin 17-O-β-D-glucopyranoside -bound to protein complex system has less residue fluctuation.

In order to assess the overall compactness of the system and its stability upon ligand binding during MD simulation, the Radius of Gyration (ROG) was calculated^100,101^. The mean Rg values for HPA-apo and HPA-limonin 17-O-β-D-glucopyranoside complex were 23.22 ± 0.08 Å and 23.17 ± 0.06 Å, respectively **(**Fig. 5C**)**. The behavior that has been observed indicates that the limonin-17-β-D-glucopyranoside molecule has a very rigid structure that opposes the HPA receptor.

By determining the protein's solvent accessible surface area (SASA), the compactness of the hydrophobic core of the protein was investigated. This was accomplished by measuring the protein's solvent-visible surface area, which is crucial for the stability of biomolecules^102^. The mean SASA values for HPA-apo and HPA- limonin 17-O-β-D-glucopyranoside complex were 16,996.92 Å and 16,749.41 Å, respectively **(**Fig. 5d**)**. The SASA finding, when paired with the observations from the RMSD, RMSF, and ROG computations, confirmed that the limonin 17-O-β-D-glucopyranoside complex system remain intact inside the catalytic domain binding site of HPA-receptor.

#### Binding interaction mechanism based on binding free energy calculation

The molecular mechanics energy technique (MM/GBSA), which combines the generalized Born and surface area continuum solvation, is a popular method for determining the free binding energies of small molecules to biological macromolecules and may be more reliable than docking scores^103^. By taking snapshots from the systems' trajectories, the binding free energies were determined using AMBER18's MM-GBSA software. Table 5 illustrates that, with the exception of ΔGsolv, all reported computed energy components produced high negative values indicating beneficial interactions. The findings showed that the HPA systems' binding affinity for limonin 17-O-β-D-glucopyranoside was -15.12 kcal/mol. The more positive Vander Waals energy components are what drive the interactions between the limonin-17-β-D-glucopyranoside compounds and the HPA receptor protein receptor residues, as shown by a thorough analysis of each individual energy contribution that yields the reported binding free energies. Significant binding free energy values, up to -37.27 kcal/mol, were observed in the gas phase for all inhibitory activities.

**Table 5:** Shows the calculated energy binding for the Limonin 17-O-β-D-glucopyranoside compound against the BCL2 receptor.

Energy Components (kcal/mol)					
Complex	ΔE_vdW_	ΔE_elec_	ΔG_gas_	ΔG_solv_	ΔG_bind_
Limonin-17-β-D-glucopyranoside –HPA	− 24.81 ± 0.68	− 4.97 ± 0.20	− 37.27 ± 1.77	22.15 ± 2.11	− 15.12 ± 0.52

∆E_vdW_ = van der Waals energy; ∆E_ele_ = electrostatic energy; ∆G_solv_ = solvation free energy; ∆G_bind_ = calculated total binding free energy.

#### Identification of the critical residues responsible for ligands binding

**Fig. 6:**
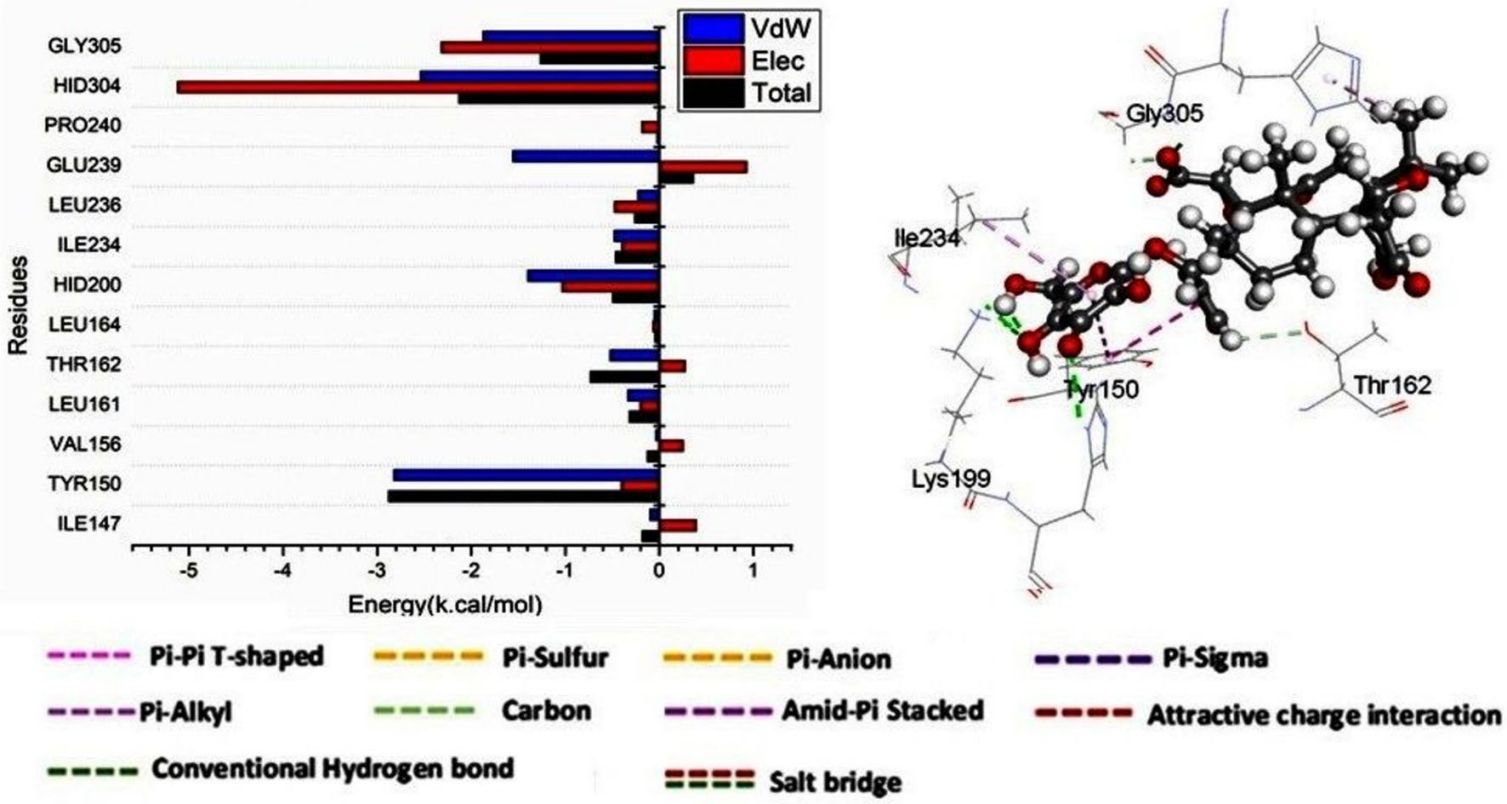
Per-residue decomposition plots showing the energy contributions to the binding and stabilization of limonin 17-O-β-D-glucopyranoside to the ATP binding site of HPA receptor.

The total energy involved when limonin 17-O-β-D-glucopyranoside compounds bind these enzymes was further decomposed into the involvement of individual site residues in order to get more knowledge about important residues involved in the inhibition of the ATP binding site of HPA receptor. From Fig. 6, the major favorable contribution of betanin compound to the ATP binding site receptor is predominantly observed from residues Tyr 150 (− 2.821 kcal/mol), Leu 161 (− 0.334 kcal/mol), Thr 162 (− 0.521 kcal/mol), Hid 200 (− 1.398 kcal/mol), Ile234 (− 0.477 kcal/mol), Leu 236 (− 0.227 kcal/mol), Glu 239 (− 1.555 kcal/mol), Hid 304 (− 2.538 kcal/mol) and Gly 305 (− 1.875 kcal/mol).

#### Ligand–residue interaction network profiles

**Fig. 7:**
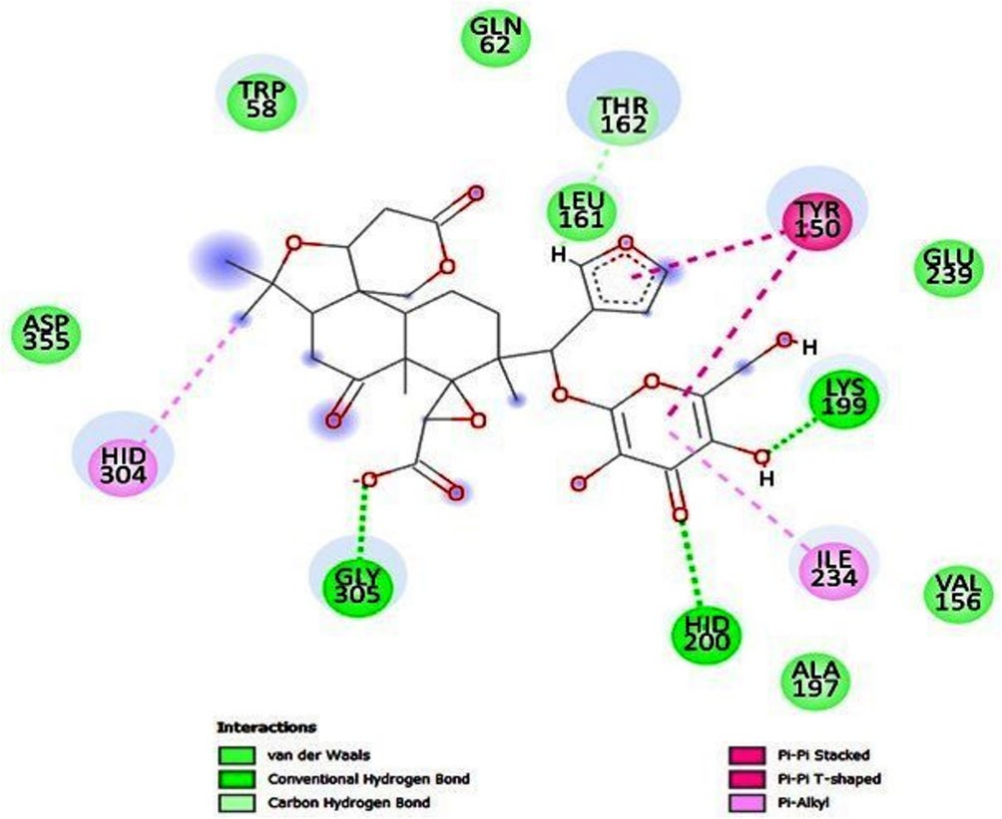
The interaction residue of limonin 17-O-β-D-glucopyranoside compound into the catalytic site of HPA receptor.

Making structural modifications to medicinal compounds in order to boost bioavailability, lower toxicity, and enhance pharmacokinetics is one goal of drug design^104^. Human pancreatic α-amylase (HPA), which acts as an accelerator for the breakdown of carbs, is one useful target for type 2 diabetes management. Blood glucose levels are reduced and hyperglycemia-related issues are mitigated by blocking α-amylase. in order for it to be regarded as a possible target for medication^105^. In the catalytic active site of HPA, molecule limonin 17-O-β-D-glucopyranoside is shown in Fig. 7. It has been discovered to form a stable hydrogen bond contact with Lys 199, HID 200, and Gly 305. Furthermore, Tyr 150 and Limonin-17-β-D-glucopyranoside have created Pi-Pi stacking.

#### Principal component analysis (PCA)

**Fig. 8:**
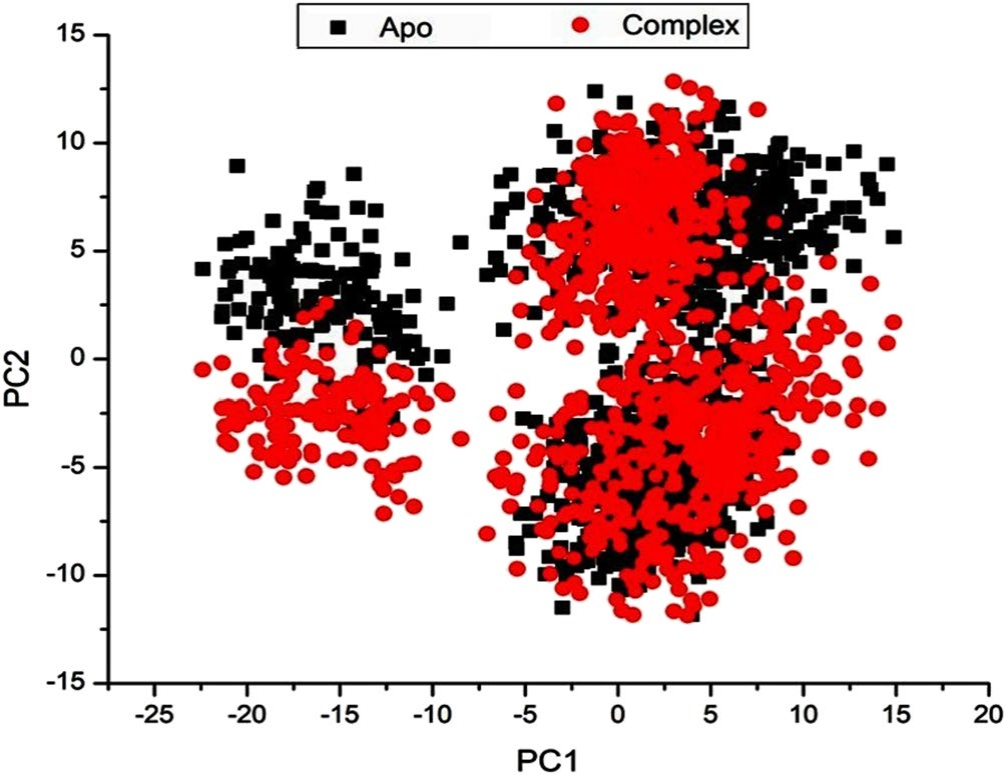
PCA projection of Cα atoms motion constructed by plotting the first two principal components (PC1 andPC2) in conformational space, apo (black), limonin 17-O-β-D-glucopyranoside (red), respectively.

The PCA plot illustrates clear and complex waves of conformation along the two major components in significant subspace, as shown in Fig. 8. The limonin 17-O-β-D-glucopyranoside and apo-protein complexes all demonstrated a substantial difference in motion; as a result, the computed eigenvector from the 20-ns MD trajectories for the three systems is very variable, demonstrating the variation in protein motion amongst the systems.

Since the apo system exhibits more atomic fluctuations than the limonin 17-O-β-D-glucopyranoside—complex system, it is likely that the ligand binding to the active sites of the protein betanin -complex system generates conformational dynamics, which is subsequently reflected by the PCs as a wave of motion. The limonin 17-O-β-D-glucopyranoside—complex system, on the other hand, is more densely packed than the other system; hence, when betanin binds to the protein, conformational flexibility is decreased, boosting ligand binding to the active site.

#### Dynamics cross-correlation matrices (DCCM) analysis

**Fig. 9:**
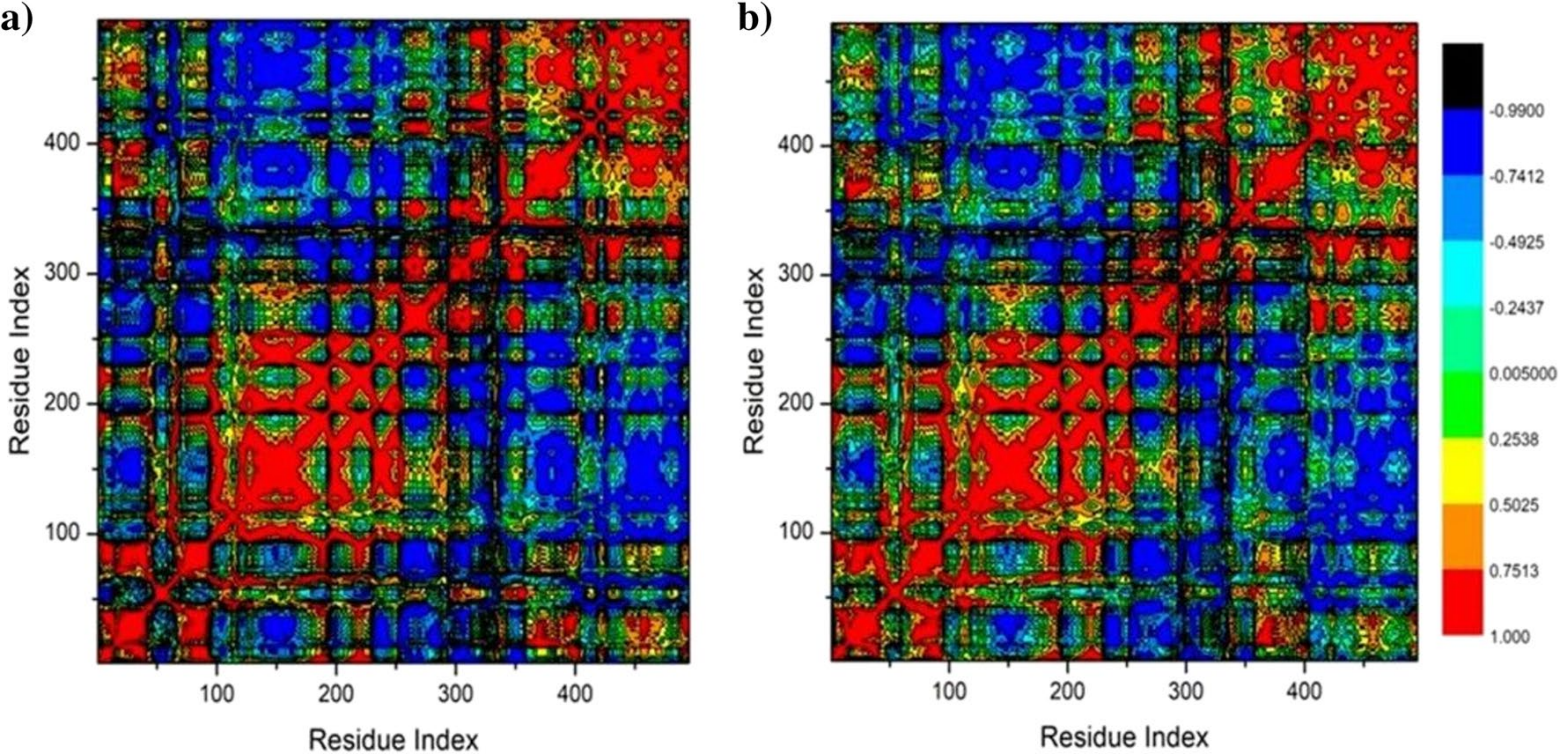
Dynamic cross-correlation matrix analyses for (**a**) Apo- HPA and (**b**) Limonin 17-O-β-D-glucopyranoside binding to HPA proteins. Numbers closer to 1 indicate high correlation, while those closer to − 1 indicate anticorrelation between pairs of residues.

In the course of the simulations, the Cα location underwent DCCM analysis to assess HPA conformational changes subsequent to ligand binding and to examine the occurrence and dynamics of linked motions **(**Fig. 5**)**. Certain residues exhibit extremely positive-correlated motions indicated by yellow–red (color) regions, whereas certain residues exhibit highly negative-correlated motions indicated by blue-black (color) regions. The systems under investigation that were assessed for this study displayed the overall correlated motions of residues in contrast to anti-correlated motions. An examination of DCCM indicates that the protein structural dynamics are provided by limonin 17-O-β-D-glucopyranoside binding to the HPA proteins. This leads to conformational changes that are reflected in differences in the related movements. Figure 9 illustrates the associated regions in the binding of limonin-17-β-D-glucopyranoside to HPA proteins. The significantly correlated region is located in residues 100–250, and residues 350–450 in the HPA protein. These areas of the receptor are the most dynamic and accept the majority of hydrophobic active site residues.

## Conclusion

The *citrus* limon peels contain limonoids that exhibit various in vitro biological activities due to their antioxidant, scavenging and anti-diabetic properties. It was found that the antioxidant (TAC and IRP), radical scavenging (DPPH, ABTS and NO) and anti-diabetic activities increased as the concentration of the samples increased (50, 100, and 200 µg/mL), indicating a concentration-dependent relationship. At the same concentration, both obacunone 17-O-β-D-glucopyranoside and limonin 17-O-β-D-glucopyranoside possessed the highest activities, followed by deacetylnomilin, and then the limonoids-rich extract. The lowest biological activities were noticed with both limonin and nomilin. The electrophoretic isoenzyme (α-amylase and α-glucosidase) patterns supported that both obacunone 17-O-β-D-glucopyranoside and limonin 17-O-β-D-glucopyranoside exhibited the highest degree in denaturing their native and SDS configuration and hence inhibiting their activities.

## Supplementary Information

Supplementary Information.

## Data Availability

All data generated or analyzed during this study is provided within the manuscript.
