# Supplementary material for: Assessment of the in vitro anti-diabetic activity with molecular dynamic simulations of limonoids isolated from Adalia lemon peels
Source: Sci Rep. 2024 Sep 14;14:21478. doi: 10.1038/s41598-024-71198-5 (PMC11401861; doi:10.1038/s41598-024-71198-5)
Supplement: Supplementary file 1 — Supplementary Information. [file 41598_2024_71198_MOESM1_ESM.docx]

**Assessment of the *in vitro* anti-diabetic activity with molecular dynamic simulations of limonoids isolated from Adalia lemon peels**

Amal M. El-Feky^a^, Wael Mahmoud Aboulthana^b,*^, Ahmed A. El-Rashedy^c^

^a^Pharmacognosy Department, Pharmaceutical and Drug Industries Research Institute, National Research Centre, 33 El Bohouth St. (Former El Tahrir St.), P.O. 12622, Dokki, Giza, Egypt.

^b^Biochemistry Department, Biotechnology Research Institute, National Research Centre, 33 El Bohouth St. (Former El Tahrir St.), P.O. 12622, Dokki, Giza, Egypt.

^c^Natural and Microbial Products Department, Pharmaceutical and Drug Industries Research Institute, National Research Centre, 33 El Bohouth St. (Former El Tahrir St.), P.O. 12622, Dokki, Giza, Egypt.

- Asst. Prof. Amal M. El-Feky

E-mail: [ammelfeky@hotmail.com](mailto:ammelfeky@hotmail.com)

- Asst. Prof. Wael Mahmoud Aboulthana (Corresponding author)

E-mail: [wmkamel83@hotmail.com](mailto:wmkamel83@hotmail.com)

- Dr. Ahmed A. El-Rashedy

E-mail: [ahmedelrashedy45@gmail.com](mailto:ahmedelrashedy45@gmail.com)

***Structure elucidation of the isolated limonoids***

**1. Compound 1 (Limonin)**


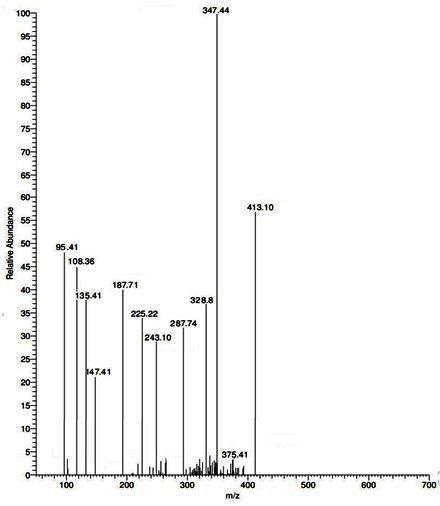


**Supplementary Fig. 1.** Mass spectrum of compound 1, limonin.


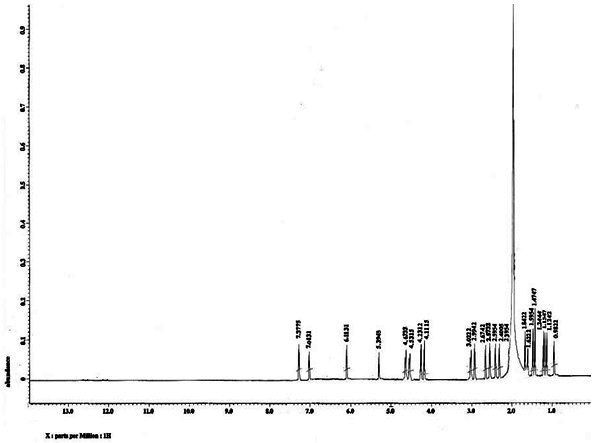


**Supplementary Fig. 2.** ^1^H-NMRspectrum of compound 1, limonin.


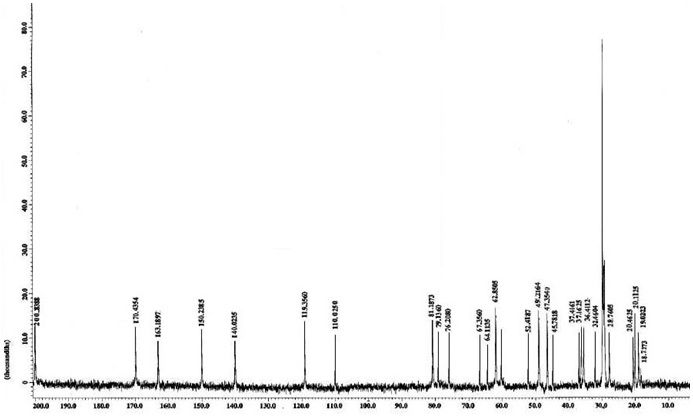


**Supplementary Fig. 3.** ^13^C-NMR spectrum of compound 1, limonin.

**Compound 2 (Deacetylnomilin)**

**
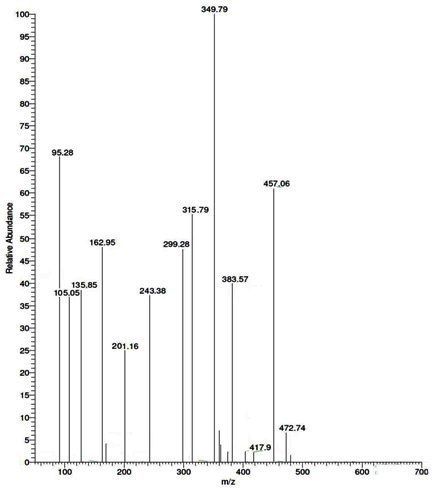
**

**Supplementary Fig. 4.** Mass spectrum of compound 2, deacetylnomilin.

**
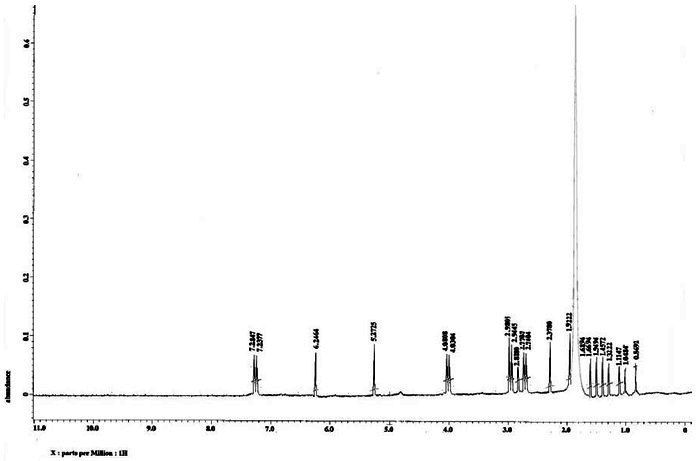
**

**Supplementary Fig. 5.** ^1^H-NMR spectrum of compound 2, deacetylnomilin.

**
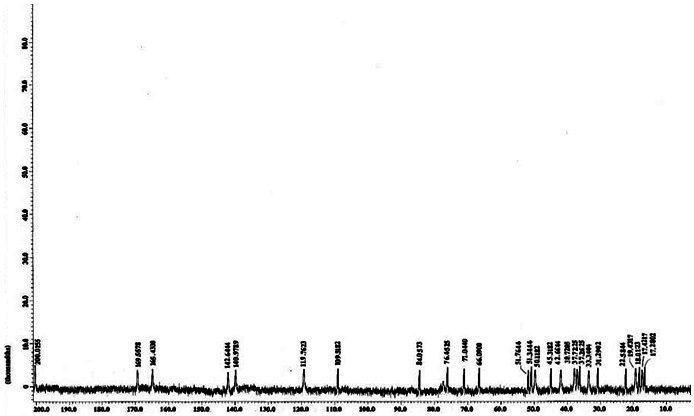
**

**Supplementary Fig. 6.** ^13^C-NMR spectrum of compound 2, deacetylnomilin

**Compound 3 (Nomilin)**

**
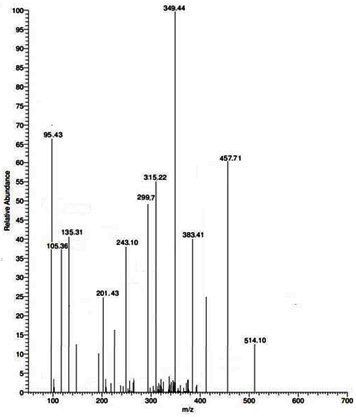
**

**Supplementary Fig. 7.** Mass spectrum of compound 3, nomilin.

**
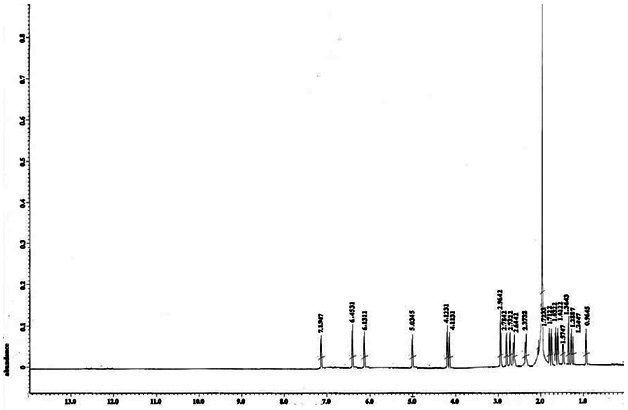
**

**Supplementary Fig. 8.** ^1^H-NMR spectrum of compound 3, nomilin.

**
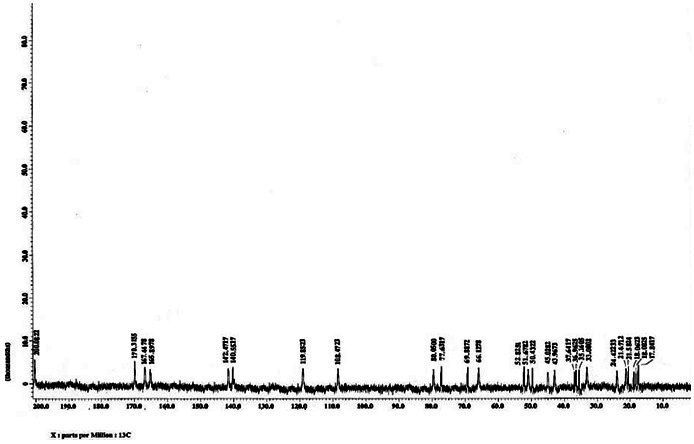
**

**Supplementary Fig. 9.** ^13^C-NMR spectrum of compound 3, nomilin

**Compound 4 (Obacunone 17-O-β-D-glucopyranoside)**

**
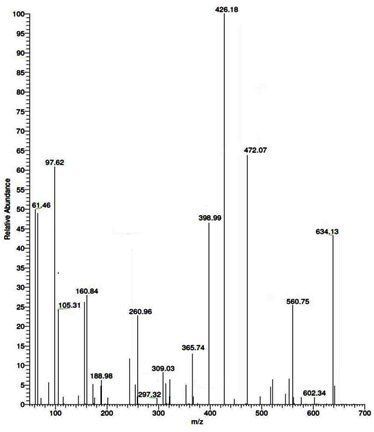
**

**Supplementary Fig. 10.** Mass spectrum of compound 4, obacunone 17-O-β-D-glucopyranoside**.**

**
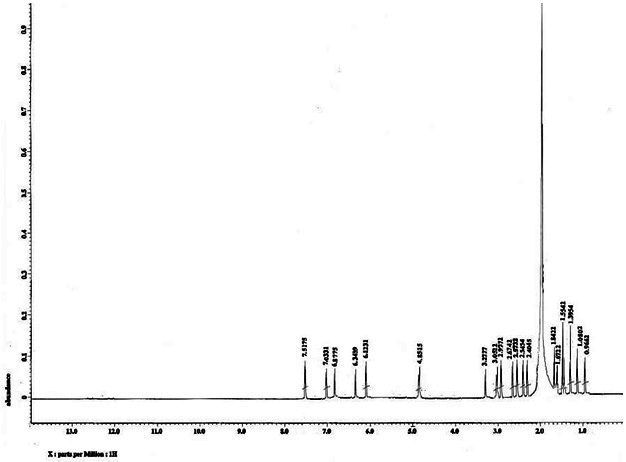
**

**Supplementary Fig. 11.** ^1^H-NMR spectrum ofcompound 4, obacunone 17-O-β-D-glucopyranoside.

**
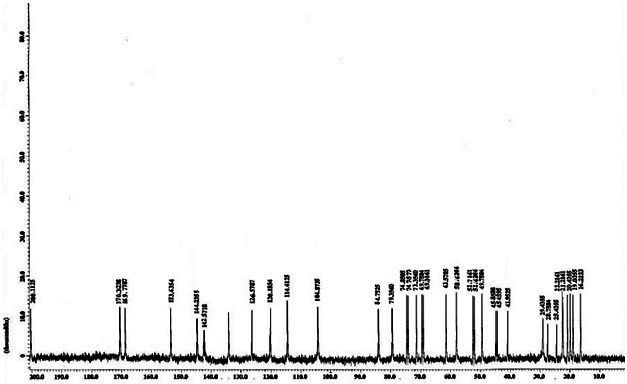
**

**Supplementary Fig. 12.** ^13^C-NMR spectrum ofcompound 4, obacunone 17-O-β-D-glucopyranoside

**Compound 5 (Limonin 17-O-β-D-glucopyranoside)**

**
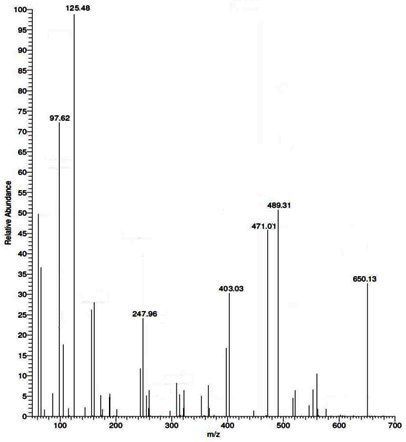
**

**Supplementary Fig. 13.** Mass spectrum of compound 5, limonin 17-O-β-D-glucopyranoside.

**
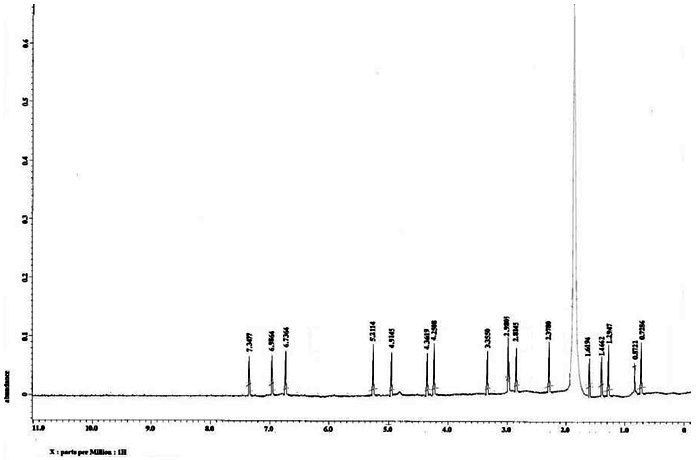
**

**Supplementary Fig. 14.** ^1^H-NMR spectrum of compound 5, limonin 17-O-β-D-glucopyranoside

**
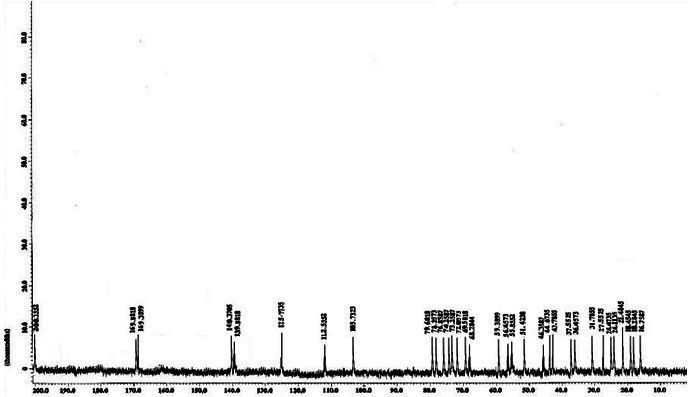
**

**Supplementary Fig. 15.** ^13^C-NMR spectrum of compound 5, limonin 17-O-β-D-glucopyranoside

***Native electrophoretic isoenzyme patterns***

***
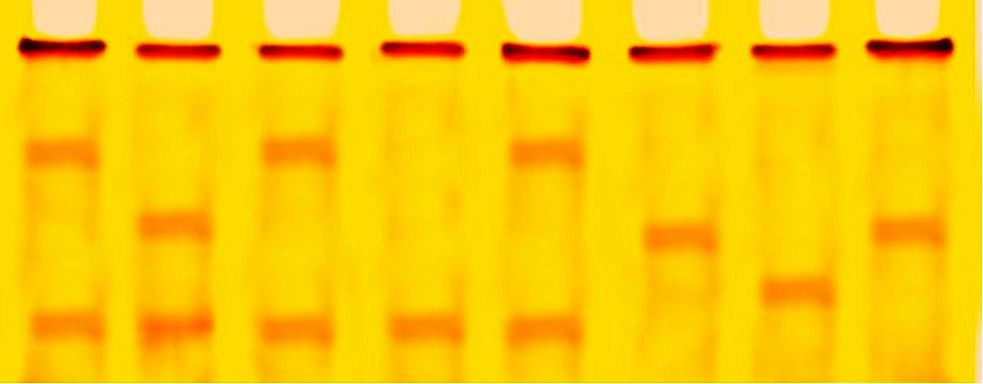
***

**Supplementary Fig. 16.** Native electrophoretic α-amylase isoenzymes pattern (the original unprocessed image) showing the anti-diabetic activity of limonoids-rich extract and the isolated compounds compared to Acarbose (standard) on the physiological state of α-amylase enzyme.

**
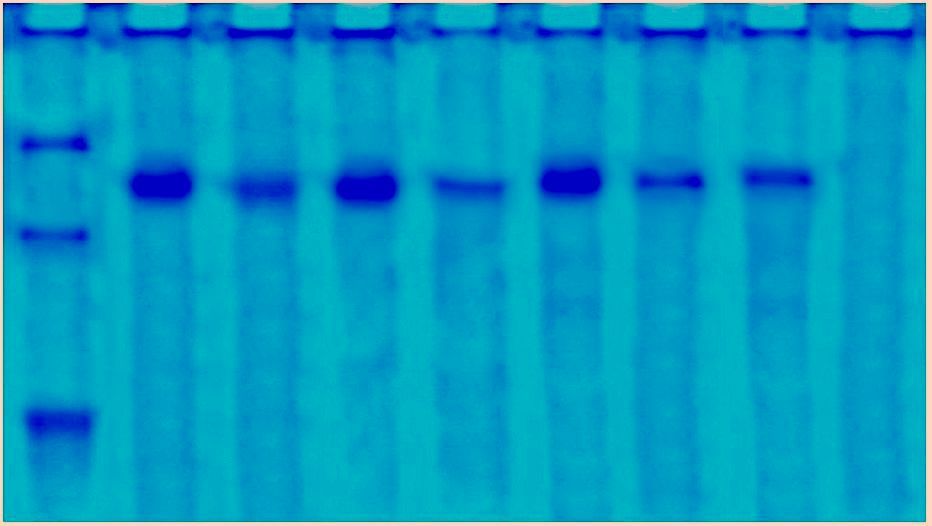
**

**Supplementary Fig. 17.** Native electrophoretic protein pattern (the original unprocessed image) showing the anti-diabetic activity of limonoids-rich extract and the isolated compounds on the quantity of the α-glucosidase enzyme checked by SDS PAGE compared to Acarbose (standard).
